# Changes in UK parental mental health symptoms over 10 months of the COVID‐19 pandemic

**DOI:** 10.1002/jcv2.12139

**Published:** 2023-03-31

**Authors:** Simona Skripkauskaite, Cathy Creswell, Adrienne Shum, Samantha Pearcey, Pete Lawrence, Helen Dodd, Polly Waite

**Affiliations:** ^1^ Department of Experimental Psychology and Department of Psychiatry University of Oxford Oxford UK; ^2^ Centre for Innovation in Mental Health School of Psychology University of Southampton Southampton UK; ^3^ School of Psychology and Clinical Language Sciences University of Reading Reading UK

**Keywords:** COVID‐19, longitudinal, mental health, pandemic, parent

## Abstract

**Background:**

The threats to health, associated restrictions and economic consequences of the COVID‐19 pandemic have been linked to increases in mental health difficulties for many. Parents, in particular, have experienced many challenges such as having to combine work with home‐schooling their children and other caring responsibilities. Yet, it remains unclear how parental mental health has changed throughout the pandemic or what factors may have mitigated or compounded the impact of the pandemic on parents' mental health.

**Methods:**

We examined monthly survey data from two linked UK‐based longitudinal studies: COVID‐19: Supporting Parents, Adolescents and Children during Epidemics' (Co‐SPACE) and COVID‐19: Supporting Parents and Young Children during Epidemics' (Co‐SPYCE). Data from 5576 parents/carers of 2–17‐year‐old children collected between April 2020 and January 2021 was analysed using mixed‐effect modelling and latent class growth (mixture) modelling.

**Results:**

Parental stress and depression, but not anxiety, were higher during the periods of restrictions. This pattern was most pronounced for parents with primary‐school‐aged children, those that worked at home or had other adults in the household. Being younger, reporting secondary or below education, working out of home, having secondary‐school‐aged children or children with special education needs (SEN)/neurodevelopmental disorders (ND) further moderated whether, how and when parental mental health symptoms changed. Although around three quarters of parents reported consistently low mental health symptoms, a substantial minority reported consistently high or increasing symptoms of anxiety, stress and depression. The latter were more likely to be parents who were younger than average, were a single adult in the household, had a pre‐existing mental health diagnosis or had a child with special educational needs or a ND.

**Conclusions:**

These findings emphasise how different personal circumstances and pre‐existing inequalities shaped how parents were affected by this unprecedented global pandemic and highlight the need for support and consideration to meet the needs of families in the future.

## Graphical abstract

Data from Co‐SPACE and Co‐SPYCE studies was examined using mixed‐effect modelling and latent class growth (mixture) modelling. Parental stress and depression, but not anxiety, were higher during the periods of restrictions. Around three quarters of parents reported consistently low symptoms. Yet, a substantial minority reported consistently high or increasing mental health symptoms, including parents who were younger, single, had a pre‐existing mental health diagnosis or had a child with SEN or a neurodevelopmental disorder.

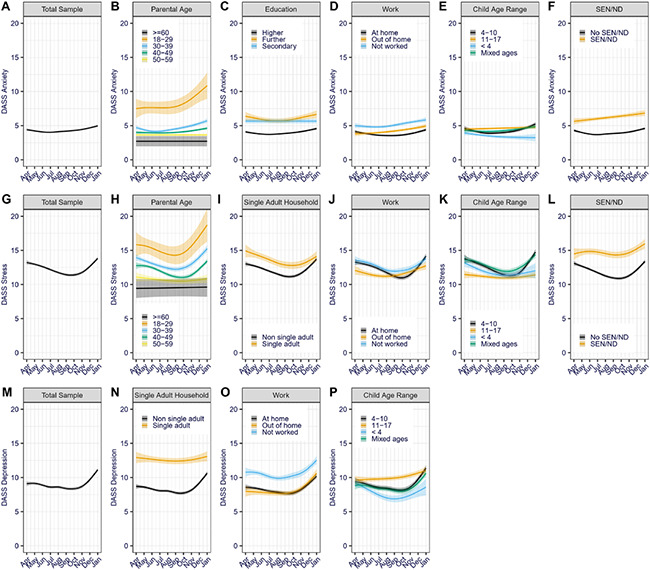

**Key points**

- Changes in parental stress and depression, but not anxiety, reflected the pattern of COVID‐19 restrictions, although most parents reported consistently low mental health symptoms.
- Some personal and family characteristics were associated with higher symptoms overall, others exacerbated symptoms when restrictions were highest and some were associated with a lack of recovery when restrictions eased.
- Parents who reported multiple risk factors were most likely to report consistently high or increasing mental health difficulties throughout the pandemic.
- The findings highlight the need for support and policy that recognises that different personal circumstances and pre‐existing inequalities shape whether, how and when parents are affected by national emergencies such as the COVID‐19 pandemic.

## INTRODUCTION

Threats to health, social restrictions and economic consequences associated with the COVID‐19 pandemic have been linked to increases in mental health difficulties among community populations (e.g., Kwong et al., 2021; Pierce et al., 2021). Psychological distress in adults appears to have been responsive to changes in restrictions following the first UK national lockdown, and this pattern has been particularly pronounced in those with children (Pierce et al., 2020). Studies have also found that maternal depression was higher during the lockdown and pandemic‐related school closures than before the pandemic (see Christie et al., 2022, for review), albeit findings regarding changes in anxiety have been less consistent (e.g., Racine et al., 2021; Wright et al., 2021).

For most parents, distress improved as restrictions eased; however not everyone will have ‘bounced back’ to previous symptom levels following repeated lockdowns and tightening of restrictions. Pierce et al. (2021) identified five distinct trajectories among UK adults between April and October 2020. Those with children were more likely to fall into the ‘recovery’ than ‘deteriorating’ or ‘consistently very poor’ groups, yet nearly a quarter of those in the latter groups also lived with children. The ‘recovery’ profile was characterised by distress that improved in July 2020, after restrictions eased and continued to improve through to October 2020, although not to pre‐pandemic levels. Indeed, research on experiences of traumatic events has shown that 25%–40% of those affected recover within the first year, many of them within 6 months, but for a small proportion effects can persist for years (Kessler et al., 2017). According to the resilience framework, whether good outcomes can be achieved in spite of serious threats to adaptation or development is dependent on an interaction of a range of vulnerability and protective factors at individual, family, community and cultural levels (Fleming & Ledogar, 2008; Masten, 2001).

It is clear that not all parents had the same experience or were affected in the same way by the pandemic and related restrictions. Some of that variability can be explained by the occurrence of the same vulnerability factors as seen in the general (adult) population. For example, being younger, having a pre‐existing mental health diagnosis, living without a partner or having lower educational attainment were repeatedly associated with greater increases in mental health difficulties at the start of the pandemic (e.g., Fancourt et al., 2020; Kwong et al., 2021; O’Connor et al., 2021; Pierce et al., 2020).

Parent‐specific disparities that have been previously shown to be independent of educational and demographic characteristics (Davis et al., 2021) are also likely to have had an effect on parental mental health throughout the pandemic. Surveys suggest that many parents faced extra challenges (Christie et al., 2022) that may have not been experienced by those without children, such as having to combine working from home with home‐schooling their children (Davis et al., 2021; Purdy et al., 2021), as well as caring for children with special education needs (SEN; Thorell et al., 2022) or neurodevelopmental (ND) conditions (Gillespie‐Smith et al., 2021). It is also likely that parental experiences would have differed based on their children's ages due to the different caretaking requirements for younger and older children. Indeed, Pierce et al. (2020) showed psychological distress in the first UK national lockdown particularly increased for those living with any younger (0–5 years‐old) rather than just older (6–15 years‐old) children. The impact of the pandemic on children's mental health may also have affected parental mental health (Luningham et al., 2022), and this may have also varied with child age. For example, parents in the UK COVID‐19: Supporting Parents, Adolescents and Children during Epidemics' (Co‐SPACE) study reported greater increases in mental health symptoms among primary school aged children, compared to secondary aged children, in the first UK national lockdown (Waite et al., 2021).

It is important to understand how and whether different vulnerability factors have affected the parent/carer population as effects on parental mental health might ‘cascade’ down and impact on a wider family system (Prime et al., 2020). Indeed, research examining family dynamics during the early months of the pandemic observed that pandemic related stress not only elevated worry, concern, sadness and stress in caregivers (Eales et al., 2021), but also increased family chaos (Cassinat et al., 2021), changed family routines and rules (Bülow et al., 2021; Eales et al., 2021) and shifted the responsibilities of childcare and education (Eales et al., 2021; Schmidt et al., 2021). These studies showed that many pandemic effects on families were disproportionally exacerbated in the presence of pre‐pandemic child and family risk factors, such as low socioeconomic status (Sun et al., 2021), mental health problems (Browne et al., 2021; Ren et al., 2021), child age (Eales et al., 2021) and lack of partner support (McRae et al., 2021). They have also shown that, in line with cumulative risk hypothesis, risk factors can have a greater effect when occurring together than they do when occurring in isolation (Fleming & Ledogar, 2008; Pereira et al., 2021).

### Current study

We examined monthly survey data from two linked UK‐based longitudinal studies. The main aim was to investigate how parent and carer self‐reported stress, anxiety and depression symptoms changed among these samples over 10 months of the pandemic (from April 2020 to January 2021), including two national lockdowns that involved most children having to stay home from school. We also set out to investigate how over time trends in parent mental health were moderated by personal and family characteristics. We expected that parental/carer anxiety, stress and depression symptoms would be higher when restrictions were high, and then would decrease as lockdown measures eased. Based on existing evidence, we also expected that there would be groups of parents/carers who would be more vulnerable to higher mental health symptoms throughout (i.e., those from a lower educational background or a single adult household, diagnosed with depression or anxiety disorders, working from home, with children with SEN, neurodevelopmental disorders [ND] and/or of primary‐school age). A secondary aim was to examine cumulative effects of vulnerability by exploring whether there were distinct trajectories associated with changes in parental/carer anxiety, stress and depression between April 2020 and January 2021, as well as what family and background characteristics predict membership within each mental health trajectory.

## METHODS

### Study design and participants

Participants responded as a part of two UK‐based, but not nationally representative, longitudinal projects^1^: Co‐SPACE (Waite et al., 2022) and ‘COVID‐19: Supporting Parents and Young Children during Epidemics’ (Co‐SPYCE; Lawrence et al., 2020). Parents and carers (hereafter known as ‘parents’) of children and young people aged between 2–4 years (Co‐SPYCE) and 4–16 years (Co‐SPACE) who lived in the UK were eligible to take part. Both projects started collecting monthly online survey data within a month of the start of the first UK national lockdown.

In total, 9805 parents took part (i.e., reached at least the questionnaire section of the survey) between 17 April 2020 and 31 January 2021. Of this group, 5842 parents completed at least one outcome subscale on at least two separate occasions. Due to missing data on predictor variables, 378 participants were removed from further analysis (for information on attrition and comparison to national figures see Tables S1 and S2). For this study, the final sample consisted of 5576 parents/carers who participated 5.45 times on average (Median = 5, Range: 2–10) with an average gap of 46.86 (Median = 32, Range: 5–275) days between the surveys, providing 23,913 observations overall.

### Procedure

Ethical approval for the studies was provided by the University of Oxford Medical Sciences Division Ethics Committee (R69060) and the University of Southampton (ERGO52617). Participants took part in the study by completing a monthly online survey on the Qualtrics platform. All participants provided informed online consent at the beginning of their first (i.e., baseline) survey, which they could complete at any point between April and December 2020 (Figure S1). A link to the follow up survey was sent via email to each parent once a month after they had completed their baseline survey. Participants could complete these follow‐up surveys at their own convenience; therefore the ‘Time’ variable represents the surveys completed at any point during the corresponding calendar month. The research protocols containing further procedural information are available via the Open Science Framework (OSF; Co‐SPACE: https://osf.io/y8ejg; Co‐SPYCE: https://osf.io/xyu6c).

### Measures

The Depression, Anxiety and Stress Scale‐21 items (DASS‐21; Antony et al., 1998; Lovibond & Lovibond, 1995) was used as a measure of parental mental health. It is a self‐report questionnaire consisting of 21 items, with 7 items per subscale: depression, anxiety and stress. Participants were asked to score every item on a scale from 0 (‘did not apply to me at all’) to 3 (‘applied to me very much’). A score for each subscale was calculated by summing the scores for the relevant items and multiplying them by 2 (range 0–42 per subscale). Internal consistency was, on average, high for anxiety (*α* = 0.84), stress (*α* = 0.92) and depression (*α* = 0.89) subscales.

Participants reported on a number of personal and family characteristics that were examined as potential predictors of parental mental health in the current study (see Supporting Information S1, pp. 6–8). This included time‐invariant sociodemographic information about themselves, such as their age and highest level of educational attainment (‘Secondary or below’, ‘Further’ or ‘Higher’), whether they had a mental health diagnosis (‘Depression/Anxiety/Other’ or ‘None’) and whether they were in a single adult household (‘Single adult’ or ‘Non single adult’), at the time of the first survey. During each survey, participants also provided time‐variant information on their working status in the past week (‘Worked at home’, ‘Worked out of the home’ or ‘Not worked’). Given it also represents the challenges of working at home, responses indicating working at both, home and out of home, were coded as ‘Worked at home’ when available. The other time‐invariant predictors included information about their children, such as the ages of the children in the household (‘Pre‐schoolers only [<4]’, ‘Primary‐school‐aged only [4–10]’, ‘Secondary‐school‐aged only [11–17]’ or ‘Mixed ages’) and whether they were reporting on a child with SEN and/or ND (‘SEN/ND’ or ‘No SEN/ND’).

### Data analysis

***How parental mental health changed over 10 months of the pandemic and how these over time trends were moderated by personal and family characteristics?***

Mixed‐effect modelling with growth curve analysis was conducted in R (version 3.6.3) using the *lmer* function in lme4 package (Bates et al., 2015). The three outcome variables (i.e., anxiety, stress and depression scores) were modelled separately. Each model consisted of a two‐level structure, where time‐invariant participant information was modelled at the second level with time‐variant repeated measures nested within. In all the models ‘Time’ variable was coded from 0 (April) to 10 (January) and participant's age was grand mean centred. In the main analyses, ‘Time’ trends, predictor variables and their interactions were simultaneously entered into the final model. This allowed us to estimate their associations with the outcome variables when controlling for other variables in the model. Maximum likelihood estimation was used in all models. The model selection process and fit indices are reported in Supporting Information (Tables S3–S5, pp. 9–12).

***Were there distinct trajectories associated with changes in parental anxiety, stress and depression over the pandemic and what characteristics predict individuals' membership within each distinct mental health trajectory?***

We used latent class growth (mixture) modelling (MPlus; Muthén & Muthén, 2000) to explore the number of distinct trajectories of parental anxiety, stress and depression over the pandemic. The best fitting number of classes was determined based on Bayesian information criterion and the Vuong‐Lo‐Mendell‐Rubin adjusted likelihood ratio (VLMR‐LRT; Lo et al., 2001; Vuong, 1989) test. Diagnostic criteria such as entropy index and smallest class size were also examined. After selection of the best model (see Table S6, p. 13), participants were classified according to their most likely group for each DASS subscale separately. Class membership was then predicted by the covariates using a multinomial logistic regression (*mlogit* package in R; Croissant, 2020) with class probability weights. For the purposes of this analysis, parents' age was recoded as a categorical variable (i.e., below or above average age). The time‐variant information on whether participants have worked at home or out of home was also recoded to represent time‐invariant categories rather than time‐variant categories used in mixed‐effect modelling. Here they were ‘worked at home’, ‘worked out of home’, ‘worked at home and out of home’, ‘not worked’ and ‘worked and not’. The obtained trajectories were compared to the reference group (defined as the largest group).

## RESULTS

The average age of the final sample was 41.20 years (standard deviation [SD] = 6.43, Range: 21–75) and most (93%) participants were mothers. Most (83%) of the sample were employed, 86% had an average household income of £16,000 or more per annum, 94% were White, and 89% resided in England. For further demographic information see Table S1. For participants' information on categorical predictor variables see Table 1. Means, SDs and correlations of continuous variables in the study can be found in Tables S7 and S8.

**TABLE 1:** Participant distribution per categorical predictor at baseline

	Final study sample (*N* = 5576)	National figures
Education		
Further	693 (12%)	12%
Higher	4513 (81%)	50%
Secondary or below	370 (7%)	38%
Single adult household		
Non single adult	4811 (86%)	85%
Single adult	765 (14%)	15%
Parent mental health diagnosis		
Depression/anxiety/other	950 (17%)	17%
No	4626 (83%)	83%
Parent work		
Not‐working	1800 (32%)	‐
Working at home	2885 (52%)	‐
Working out of home	891 (16%)	‐
Children's age		
Pre‐schoolers (<4‐year‐old) only	672 (12%)	21%
Children (4–10‐year‐old) only	1887 (34%)	41%
Adolescents (11–17‐year‐old) only	1147 (21%)	38%
Mixed ages	1870 (34%)	‐
Child SEN/ND status		
No SEN/ND	4797 (86%)	84%
SEN/ND	779 (14%)	16%

*Note*: ‘Baseline’ refers to the participants' first survey (Figure S1). ‘Mixed ages’ refers to parents with children falling into multiple age categories (e.g., pre‐schoolers and adolescents). SEN/ND refers to the child's special education needs or neurodevelopmental disorders. See Tables S1 and S2 for additional details on national figures.

***How parental mental health changed over 10 months of the pandemic and how these over time trends were moderated by personal and family characteristics?***

**FIGURE 1:**
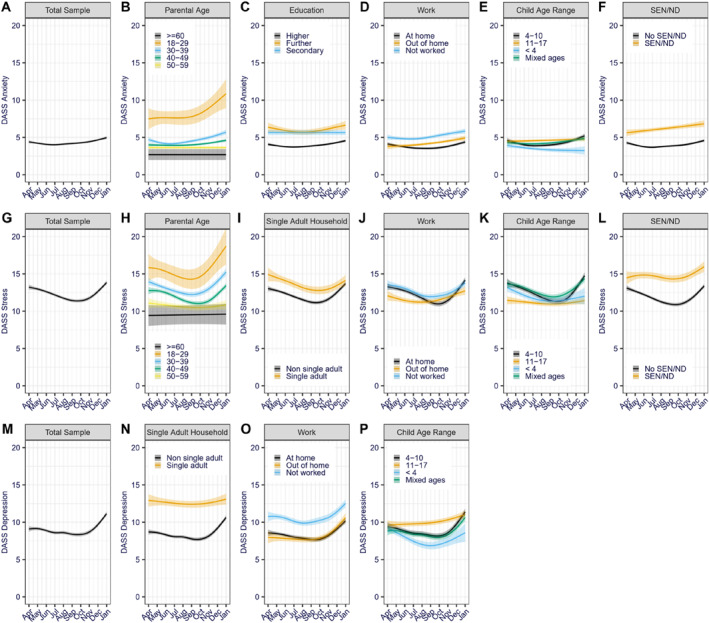
Predicted anxiety (A–F), stress (G–L) and depression (M–P) growth trajectories over time per significant moderators. Shaded area represents 95% confidence intervals. Only terms for significant interactions in the final models are depicted (see Table 2 for coefficients of the non‐significant interactions).

Estimated parameters for final mixed‐effect models across the three outcome variables are presented in Table 2. All the predictor variables were differentially associated with the averages of mental health scores in at least one of the three final models. Significant predictors of slope are presented in Figure 1. The summary of the main effects is provided in the Supporting S1 (p. 16).

**TABLE 2:** Estimated effects and their standard errors (SE) for final models and secondary (stressor) analysis per outcome measure

	Anxiety		Stress		Depression	
	*b*	SE	*b*	SE	*b*	SE
T1	0.35*	0.16	−0.37	0.24	0.76*	0.24
T2	1.17**	0.16	3.16**	0.24	2.30**	0.21
T3			1.86**	0.23	1.02**	0.12
Parental age	−0.12**	0.01	−0.12**	0.02	−0.06*	0.02
Edu: Further (vs. higher)	0.91**	0.21	−0.26	0.31	0.97*	0.31
Edu: <Secondary (vs. higher)	0.99**	0.28	−1.07*	0.41	0.81	0.41
Single adult	1.37**	0.20	0.62*	0.30	2.68**	0.30
MH diagnosis	5.31**	0.18	6.68**	0.27	6.78**	0.27
Work: Out of home (vs. home)	0.01	0.09	−0.45*	0.14	−0.64**	0.13
Work: Not worked (vs. home)	0.05	0.08	−0.69**	0.13	0.41*	0.12
Ch. ages: 11–17 (vs. 4–10)	0.61*	0.21	−1.14**	0.30	0.63*	0.31
Ch. ages: <4 (vs. 4–10)	−1.08**	0.25	−0.94*	0.36	−0.84*	0.37
Ch. ages: Mixed (vs. 4–10)	−0.03	0.17	0.07	0.24	−0.26	0.25
SEN/ND	1.51**	0.20	2.50**	0.29	2.23**	0.30
T1*Parental age	−0.04*	0.02	−0.05*	0.02	−0.05	0.02
T2*Parental age	−0.02	0.01	−0.03	0.02	−0.04	0.02
T1*Edu: Further (vs. higher)	0.40	0.29	0.10	0.42		
T1*Edu: <Secondary (vs. higher)	0.04	0.41	0.60	0.61		
T2*Edu: Further (vs. higher)	0.08	0.26	−0.34	0.39		
T2*Edu: <Secondary (vs. higher)	−0.78*	0.35	−0.83	0.53		
T3*Edu: Further (vs. higher)			−0.62	0.39		
T3*Edu: <Secondary (vs. higher)			−0.86	0.54		
T1*Single adult			−0.06	0.38	−0.24	0.38
T2*Single adult			−0.70*	0.36	−0.91*	0.36
T1*Work: Out of home (vs. home)	0.66*	0.24	1.32**	0.36	1.06*	0.35
T1*Work: Not worked (vs. home)	0.38	0.21	0.18	0.30	−0.07	0.29
T2*Work: Out of home (vs. home)	−0.11	0.23	−0.61	0.34		
T2*Work: Not worked (vs. home)	−0.41*	0.19	−0.62*	0.29		
T3*Work: Out of home (vs. home)			−1.05*	0.33		
T3*Work: Not worked (vs. home)			−0.27	0.29		
T1*Ch. ages: 11–17 (vs. 4–10)	0.16	0.26	0.94*	0.38	0.96*	0.38
T1*Ch. ages: <4 (vs. 4–10)	−1.03*	0.39	−0.62	0.60	−0.44	0.57
T1*Ch. ages: Mixed (vs. 4–10)	−0.19	0.20	−0.01	0.30	−0.17	0.30
T2*Ch. ages: 11–17 (vs. 4–10)	−0.79*	0.24	−1.57**	0.36	−0.74*	0.36
T2*Ch. ages: <4 (vs. 4–10)	−0.34	0.34	−1.05*	0.53	0.48	0.51
T2*Ch. ages: Mixed (vs. 4–10)	−0.73**	0.19	−0.55	0.29	−0.45	0.29
T3*Ch. ages: 11–17 (vs. 4–10)			−0.75*	0.34		
T3*Ch. ages: <4 (vs. 4–10)			−1.36	0.46		
T3*Ch. ages: Mixed (vs. 4–10)			−0.26	0.28		
T1*SEN/ND	1.15**	0.24	0.96*	0.36	0.62	0.36
T2*SEN/ND			−0.31	0.34	−0.40	0.34

Abbreviations: Ch. ages, children's ages in the household; Edu., highest level of parental education; MH diagnosis, previous parental mental health diagnosis; SEN/ND, child's special education needs or neurodevelopmental disorders; T1, linear time trend; T2, quadratic time trend; T3, cubic time trend.

*p < 0.05; **p < 0.001.

*Changes in parental anxiety*. The growth modelling of parental anxiety symptoms over time identified both linear and quadratic trends (Figure 1A). In other words, anxiety symptoms generally increased over time, but the month‐to‐month change in symptoms was higher in later than earlier months. Many of the personal and family characteristics moderated how anxiety symptoms changed over the period examined. For instance, being a younger than average parent (Figure 1B), working out of home rather than at home (Figure 1D) or having a child with SEN/ND (Figure 1F) were associated with a greater linear increase in anxiety symptoms between April 2020 and January 2021. Having only pre‐school‐aged children (<4‐year‐old) also moderated the linear development of anxiety symptoms (Figure 1E) over time; whilst anxiety symptoms between April 2020 and January 2021 increased in parents of 4–10‐year‐olds, they decreased in parents of under 4‐year‐olds. Having children who were only 11–17 years old or having children of mixed ages moderated the quadratic trend in anxiety symptoms over time; as can be seen in Figure 1E, having children in these age ranges indicated a less curved change over time in comparison to those with 4–10‐year‐olds. In other words, whilst the month‐to‐month anxiety increase was higher in later than earlier months for parents of 4–10‐year‐olds, anxiety symptoms increased in parents of 11–17‐year‐olds and decreased in parents of under 4‐year‐old in a more consistent month‐to‐month rate than in parents of 4–10‐year‐olds. Similarly, reporting secondary or below education (Figure 1C) or not working (Figure 1D) moderated the quadratic change in anxiety symptoms over time, also indicating a flatter curve.

*Changes in parental stress*. The growth modelling of parental stress symptoms over time identified significant quadratic and cubic trends for stress (Figure 1G); stress scores decreased between April and September 2020 and then increased between October 2020 and January 2021 to the same level as observed in April 2020. This pattern of stress symptoms over time was further moderated by some of the personal and family characteristics. Specifically, being a younger than average parent (Figure 1H), working out of home rather than at home (Figure 1J), having children aged 11–17 (rather than 4–10) years only (Figure 1K), or having a child with SEN/ND (Figure 1L) were all associated with a higher linear increase in stress symptoms between April 2020 and January 2021. Having only 11–17‐year‐olds also moderated the quadratic and cubic trends and having only a pre‐school‐aged child or children moderated the quadratic trend, both indicating a less curved change in stress symptoms over time compared to parents of 4–10‐year‐old children (Figure 1K). In other words, for parents of 4–10‐year‐olds stress scores decreased more between April and September 2020 and then increased more between October 2020 and January 2021 compared to parents of younger or older offspring. Similarly, being a single adult in the household (Figure 1I) or not working (Figure 1J) also moderated the quadratic trend in stress symptoms, whilst working away from home moderated the cubic change in stress symptoms—both indicating a less curved change in symptoms over time.

*Changes in parental depression*. The growth modelling of parental depression symptoms over time identified significant linear, quadratic and cubic trends (Figure 1M); parental depression symptoms decreased from around May to September/October 2020 and then increased again through to January 2021 resulting in an overall linear increase in depression symptoms over time. Compared to anxiety and stress symptoms, fewer of the personal and family characteristics moderated this pattern of change. Specifically, only working out of home (compared to working at home; Figure 1O) or having children aged 11–17 years (compared to 4–10‐year‐olds; Figure 1P) predicted a greater linear increase in depression symptoms between April 2020 and January 2021. Having children who were all 11–17 years old (Figure 1P), as well being a single adult in the household (Figure 1N), moderated the quadratic trend in depression symptoms over time resulting in flatter, but still increasing, curve of change over time in these groups.

### Secondary analysis

***Were there distinct trajectories associated with changes in parental anxiety, stress and depression over the pandemic and what characteristics predict individuals' membership within each distinct mental health trajectory?***

After fitting models with two to five latent classes (Table S6), the three‐class model yielded the best fit for parental anxiety and five‐class models were considered the best fit for parental stress and depression (for final model estimates see Table S9).

**FIGURE 2:**
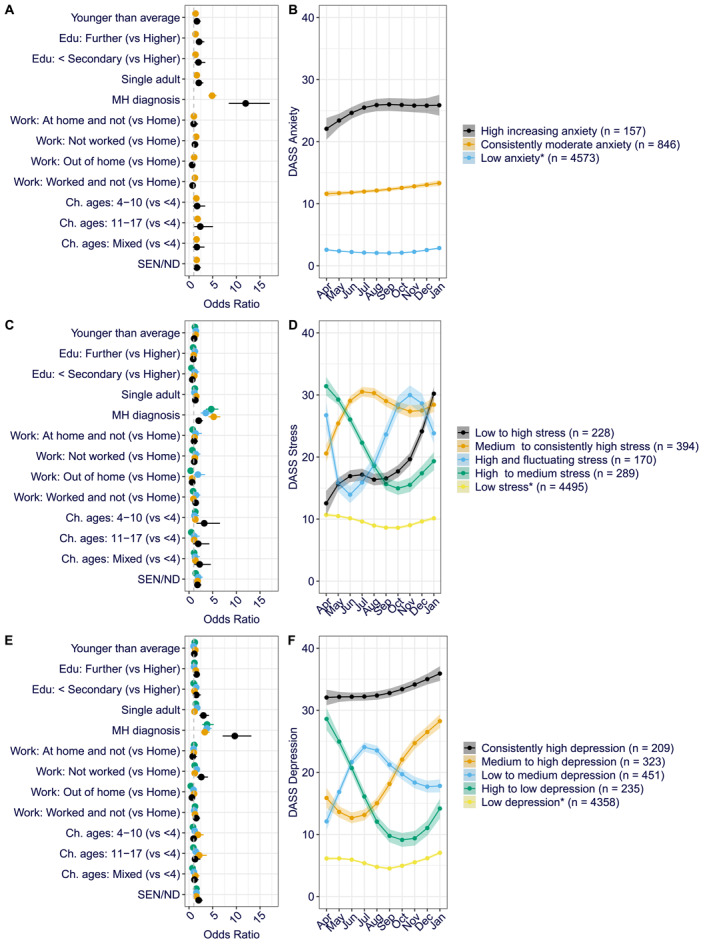
Odd ratios and latent class trajectories for parental anxiety (A, B), stress (C, D) and depression (E, F). *Reference group. Error bars and shaded areas represent 95% confidence intervals.

*Trajectories of parental anxiety* (Figure 2A,B). Most parents (82%) were classified as being in the ‘low anxiety’ group. The rest were mostly (15%) classified as the ‘consistently moderate’ group. However, a small proportion (3%) of parents were classified as the ‘high increasing anxiety’ group characterised by a sharp increase in anxiety during the first lockdown followed by relatively stable symptoms.

Parents in the ‘consistently moderate anxiety’ group, in comparison to the ‘low anxiety group’, were more likely to be younger than average (odds ratio [OR] = 1.37), have completed further (OR = 1.38) or secondary or below (OR = 1.37) education, be a single adult in the household (OR = 1.64), have a pre‐existing mental health diagnosis (OR = 4.89), have children over 4‐years‐old (4–10: OR = 1.54; 11–17: OR = 1.80, Mixed ages: OR = 1.58) or with SEN/ND (OR = 1.62). They were also more likely to not have worked throughout the whole study period (OR = 1.51) or at some points (OR = 1.25). Those in the ‘high increasing anxiety’ group were also more likely than the ‘low anxiety group’ to be younger than average (OR = 1.66), have completed further (OR = 2.13) or secondary or below (OR = 2.00) education, be a single adult in the household (OR = 2.06), have 11–17‐year‐old children (OR = 2.38), children with SEN/ND (OR = 1.65) and particularly have a pre‐existing mental health diagnosis (OR = 11.97). Full characteristics of the three anxiety trajectories are shown in Tables S10and S11.

*Trajectories of parental stress* (Figure 2C,D). Most parents (81%) were also most likely to be in the ‘low stress’ group. A second group (4%) had low stress scores at the beginning of the pandemic but increasing stress symptoms throughout the following 10 months (‘low to high’ group). A third group, characterised by an increase in stress symptoms, consisted of 7% of parents; they had medium levels of stress symptoms that increased over the early lockdown and remained stable and high since (‘medium to consistently high stress’ group). A fourth group (5%) showed high stress scores at the beginning of the pandemic that decreased over time before somewhat increasing again when new restrictions were introduced in December 2020–January 2021 (‘high to medium stress’ group). The fifth and final trajectory of stress symptoms characterised the 3% of parents. They showed high stress symptoms initially but a decrease in stress symptoms during the first lockdown followed by an increase in reported stress over the summer and autumn months when restrictions were eased and a further decrease when winter lockdown was introduced (‘high and fluctuating’ group).

Full characteristics of the five stress trajectories are shown in Tables S12 and S13. Parents in the ‘low to high stress’ group were more likely than those in the ‘low stress’ group to have a mental health diagnosis (OR = 4.70), have primary‐school‐aged children (4–10: OR = 3.22; Mixed ages: OR = 2.25) or have children with SEN/ND (OR = 1.79). The ‘medium to consistently high stress’ group was characterised by being younger than average (OR = 1.41), being a single adult in the household (OR = 1.52), having a mental health diagnosis (OR = 5.21) or having children with SEN/ND (OR = 1.81). The ‘high to medium stress’ group was more likely than the ‘low stress’ group to have a mental health diagnosis (OR = 4.70), but less likely to have worked out of home throughout the pandemic (OR = 0.35) or have older children (11–17: OR = 0.47). As with the other higher stress groups, the ‘high and fluctuating’ group was also characterised by having a mental health diagnosis themselves (OR = 3.54) and having children with SEN/ND (OR = 1.93). However, unlike other higher stress groups, these parents were more likely to have worked out of the home throughout the pandemic (OR = 1.88).

*Trajectories of parental depression* (Figure 2E,F). Most parents in our sample (78%) were classified in the ‘low depression’ group. A second group (8%) showed low depression symptoms at the beginning of the pandemic, an increase over the first lockdown and then a slow decrease again as restrictions eased (‘low to medium depression’ group). A third group (6%) also started the pandemic with relatively low depression scores, which decreased further during the first lockdown but then increased (‘medium to high depression’ group). Two further groups of parents had high depression scores at the beginning of the pandemic. For 4% of parents, these initially high depression scores decreased over the first lockdown and subsequent easing of restrictions but started increasing again when new restrictions were introduced in December 2020 and January 2021 (‘high to low depression’ group). The final group, which included 6% of parents, had consistently high depression scores (‘high depression’ group).

Full characteristics of the five depression trajectories are shown in Tables S14 and S15. The ‘low to medium depression’ group was more likely than the relatively consistent ‘low depression’ group to have completed secondary or below education (OR = 1.52), be the single adult in the household (OR = 1.73), have a mental health diagnosis (OR = 3.78), have not worked throughout the pandemic (OR = 1.64), and have children with SEN/ND (OR = 1.57). The ‘medium to high depression’ group was characterised by being a younger than average parent (OR = 1.32), having completed further education (OR = 1.44), having mental health diagnosis (OR = 3.33), having primary‐school‐aged children (4–10: OR = 1.89; 11–17: OR = 2.16) and children with SEN/ND (OR = 1.55). The ‘high to low depression’ group was more likely than the ‘low depression’ group to have a mental health diagnosis (OR = 3.81) or have children with SEN/ND (OR = 1.55), but less likely to have worked out of the home throughout the pandemic (OR = 0.37). Parents in the ‘high depression’ group were likely to have completed further education (OR = 1.59), be the single adult in the household (OR = 3.04), have mental health diagnosis (OR = 9.67), have not worked throughout the pandemic (OR = 2.60) and have children with SEN/ND (OR = 2.04).

## DISCUSSION

The current study examined how anxiety, stress and depression symptoms changed among UK parents in the Co‐SPACE and Co‐SPYCE studies between April 2020 and January 2021 during the COVID‐19 pandemic. First, we found that parental stress and depression, but less so anxiety, followed the pattern of restrictions. Different personal circumstances and pre‐existing inequalities moderated whether, how and when parental mental health symptoms changed. Generally, parents of secondary‐school‐aged children (aged 11–17 years) or those working out of the home did not exhibit an immediate increase in mental health symptoms during the first national lockdown but showed a greater continuous increase in mental health symptoms over time without a pattern of recovery. Being a younger parent or having a child with SEN/ND predicted greater overall mental health symptoms that were further exacerbated during the pandemic but, similarly to the average pattern, increased and decreased in line with restrictions. Those parents who reported having secondary‐level or below education or being a single adult in a household also reported greater overall symptoms and although their increase was similar to that of the average pattern, they showed no recovery when restrictions eased. Second, we identified distinct trajectories of parental anxiety, stress and depression over the pandemic. While around three quarters of parents reported low anxiety, stress or depression, a substantial minority (3%–18%) reported consistently high or increasing symptoms of anxiety, stress and depression. When cumulative effects were examined, those with high or increasing symptoms were more likely to be parents who were younger than average, were a single adult in the household, had a pre‐existing mental health diagnosis, and had one or more children with SEN/ND.

Our finding that parental stress and depression were at their worst when restrictions were high and improved as restrictions eased is consistent with previous research that found elevated parental mental health symptoms early in the pandemic, when restrictions were strict, compared to pre‐pandemic data (Racine et al., 2021; Wright et al., 2021) and with data from the general adult populations in the UK showing that the mental health symptoms improved as restrictions eased in the summer 2020 (Fancourt et al., 2020; Pierce et al., 2021). It was notable that both stress and depression started increasing again in autumn through to January 2021 as new restrictions were introduced, with parental depression surpassing the symptom levels reported in April 2020. This is consistent with findings from cross‐sectional surveys (see Christie et al., 2022) and qualitative interviews (Shum et al., 2023) with parents around the same time who reported feelings of mental exhaustion as they attempted to juggle parenting and home‐schooling with other responsibilities. Interestingly, in our study, anxiety symptoms followed a different pattern with an accelerating rate of increase over time. As with depression, anxiety scores in the second national lockdown surpassed those in the first. This is also consistent with qualitative data where parents highlighted the ongoing uncertainty and worry as restrictions were reintroduced (Shum et al., 2023).

We identified several characteristics that predicted overall worsening mental health over the 10‐month period that were consistent with previous parenting research suggesting that both parent and child level factors are likely to influence parental responses to stressors (Crnic & Low, 2002). In line with previous research conducted during the pandemic, being a younger parent (Pierce et al., 2020) and having a child with SEN/ND (Gillespie‐Smith et al., 2021; Thorell et al., 2022) were associated with both high average levels and increases in mental health symptoms beyond the average change. Previous research shows that the pandemic has resulted in changed family routines and rules (Bülow et al., 2021; Eales et al., 2021) and shifted the responsibilities of childcare and education (Eales et al., 2021; Schmidt et al., 2021). These effects may have been felt particularly strongly by younger parents and those with children with SEN/ND who may have previously depended on support by extended family and support services. Consistent with previous research (Purdy et al., 2021), working out of the home, whilst on average indicating lower levels of mental health symptoms than working at home, also predicted overall worsening mental health. It is possible that parents who needed to work out of the home were less overwhelmed by home‐schooling demands but were more likely to be keyworkers and thus experience greater worry and perceived risk of COVID‐19 (Ayling et al., 2020). Having primary‐school‐aged children predicted higher average mental health symptoms, relative to those with secondary or pre‐school aged children, that followed the pattern of restrictions. This may reflect the demands of both caretaking and supporting home schooling. Also of note, this mirrored the pattern of mental health symptoms seen in the children of the same sample (Creswell et al., 2021) in line with previous evidence suggesting a bidirectional relationship between parental and child mental health (Luningham et al., 2022). Unlike Pierce et al. (2020) who found particularly elevated psychological distress symptoms among parents whose youngest child was under 5 years old in the first UK national lockdown, we found that having only pre‐school‐aged children (<4 years) was associated with *decreasing* anxiety scores. It is possible, however, that this reflected differences in how the two studies categorised child age, with Pierce et al. (2020) categorising parents by the age of the youngest child and grouping all school‐aged (6–15 years old) children together, potentially masking specific patterns among families of pre‐school‐, primary‐ and secondary‐school‐aged children. Of note, parents of secondary‐school‐aged children (aged 11–17 years) exhibited a potentially additive effect of lockdowns and restrictions with greater continuous increases in mental health symptoms from the start of the pandemic. This increase in mental health symptoms in parents of adolescent children may reflect stress and changes in mood caused by the impact that the pandemic had on adolescents themselves; including disruptions to sleep, mood and quality of relationships (Illingworth et al., 2022) as well as ongoing concerns about disruption, delays and uncertainty in education and recreational activities at a key point in the young person's life that may have a significant bearing on their future (Pearcey et al., 2022).

In line with the findings by Pierce et al. (2021), well‐established inequalities in mental health prior to the pandemic were maintained when other predictors were accounted for. For parents with pre‐existing mental health diagnoses and lower education backgrounds, stress and depression increased and decreased in line with restrictions. Although we did not find a significant additional increase in mental health symptoms, they were, on average, at a relatively high level throughout. In the case of single adult households, stress and depression remained high throughout the 10 months. When looked at in isolation, this group showed little improvement when restrictions eased. Furthermore, along with parents with pre‐existing mental health diagnosis, from lower education background, who were younger or had a child with SEN/ND, parents in single adult households were consistently more likely to be in the small but most disadvantaged groups showing deteriorating mental health across anxiety, stress and depression symptoms. This finding is consistent with the cumulative risk hypothesis which suggests that risk factors can have a greater effect when occurring together than they do when occurring in isolation (Fleming & Ledogar, 2008; Pereira et al., 2021).

### Limitations and future directions

This study provides a unique insight into month‐to‐month changes in parental mental health through the first 10 months of the COVID‐19 pandemic. However, it is important to be aware of the limitations. For instance, only 56.9% of the full sample provided information on the outcome and predictor variables at least twice in the period examined (Tables S1 and S2) and thus were included in the current study. Whilst the resulting sample was large enough to conduct the current analyses, it is not known whether the observed patterns of change would be generalisable to those who dropped out of the study after participating just once. As the Co‐SPACE and Co‐SPYCE projects were started in response to the COVID‐19 pandemic, this study does not include any data from the first 3 weeks of the first national UK lockdown or from the pre‐pandemic. This means that we cannot draw conclusions about the immediate impact of the pandemic on parental mental health. There are also other factors (not included in the current study) that could further explain the heterogeneity observed here. It will be important to further understand the impact of time‐variant economic, familial and interpersonal changes on parental stress and mental health during the pandemic.

Numerous studies have now shown deteriorating mental health in parents compared to pre‐pandemic levels (Pierce et al., 2020; Racine et al., 2021; Wright et al., 2021), suggesting that it is unlikely that mental health changes observed in the current study were fully independent from the context of the COVID‐19 pandemic. Yet, we cannot rule out the possibility that changes in parent mental health over time relate to other non‐pandemic factors, such as increasing academic demands on their children with age (e.g., Branje et al., 2012) or seasonal changes in mood (Harmatz et al., 2000; Murray et al., 2001). Daly et al. (2020) evidenced that the role of seasonality in change of adult psychological distress symptoms observed in April to June 2020 was minimal when compared to pre‐pandemic data in the nationally representative United Kingdom Household Longitudinal Study. Nevertheless, to reliably determine the effects of seasonality, different restrictions, case numbers and vaccination rates, future studies would benefit from comparing changes in parental mental health between countries following different pandemic timelines.

## CONCLUSIONS

While the majority of parents in the current sample experienced relatively stable and low levels of stress, anxiety and depression symptoms throughout the first 10 months of the pandemic in the UK, certain family and personal characteristics conferred particular vulnerability for either mental health deterioration. These findings emphasise how different personal circumstances and pre‐existing inequalities have shaped how parents and families have been affected during this unprecedented global pandemic and highlight the need for support and consideration to meet the needs of parents and families going forwards.

## AUTHOR CONTRIBUTIONS

**Simona Skripkauskaite**: Conceptualization; Data curation; Formal analysis; Investigation; Methodology; Software; Visualization; Writing – original draft; Writing – review & editing. **Cathy Creswell**: Conceptualization; Funding acquisition; Project administration; Writing – review & editing. **Adrienne Shum**: Data curation; Investigation; Software; Writing – review & editing. **Samantha Pearcey**: Data curation; Investigation; Software. **Pete Lawrence**: Conceptualization; Funding acquisition; Investigation. **Helen Dodd**: Conceptualization; Funding acquisition; Writing – review & editing. **Polly Waite**: Conceptualization; Funding acquisition; Project administration; Writing – review & editing.

## CONFLICT OF INTEREST

Polly Waite is funded by an NIHR Postdoctoral Research Fellowship (PDF‐2016‐09‐092). Cathy Creswell is supported by the Oxford and Thames Valley NIHR Applied Research Collaboration. Helen Dodd is supported by a UKRI Future Leaders Fellowship (MR/S017909/1). The views expressed in this publication are those of the authors and not necessarily those of the NIHR or the Department of Health and Social Care. The remaining authors have declared that they have no competing or potential conflicts of interest.

## ETHICAL CONSIDERATIONS

Ethical approval for the studies was provided by the University of Oxford Medical Sciences Division Ethics Committee (R69060) and the University of Southampton (ERGO52617).

## Supporting information

Supporting Information S1

## Data Availability

The full data that support the findings of this study are available on request from the corresponding author due to privacy restrictions. The Co‐SPACE data are partially available under safeguarded access via the UK Data Service at http://doi.org/10.5255/UKDA‐SN‐8900‐1, reference number SN 8900.

## ENDNOTE

^1^
The primary statistical analysis of the Co‐SPACE data was pre‐registered via Open Science Framework (OSF; https://osf.io/vr3wj ). The final analysis was extended to include the Co‐SPYCE sample enabling us to also evaluate mental health symptoms in parents of pre‐schoolers. It was further extended to include a secondary analysis using latent class growth (mixture) modelling.
