# Supplementary material for: Changes in UK parental mental health symptoms over 10 months of the COVID‐19 pandemic
Source: JCPP Adv. 2023 Mar 31;3(2):e12139. doi: 10.1002/jcv2.12139 (PMC10519732; doi:10.1002/jcv2.12139)
Supplement: Supplementary file 1 — Supporting Information S1 [file JCV2-3-e12139-s001.docx]

Supporting Information

Table S1

Demographic characteristics (at Baseline) of the final sample in comparison to the excluded participants and national figures

|  | Initial sample | Attrition | | | Final sample | National figures |
| --- | --- | --- | --- | --- | --- | --- |
|  |  | Missing outcomes | Single participation | Missing predictors |  |  |
|  | (*N*=9805) | (*N*=931) | (*N*=3032) | (*N*=378) | (*N*=5576) |  |
| Relationship to child |  |  |  |  |  |  |
| Mother | 8760 (89.3%) | 700 (75.2%) | 2696 (88.9%) | 275 (72.8%) | 5197 (93.2%) | - |
| Father | 684 (7.0%) | 76 (8.2%) | 238 (7.8%) | 35 (9.3%) | 341 (6.1%) | - |
| Grandfather | 5 (0.1%) | 0 (0.0%) | 2 (0.1%) | 0 (0.0%) | 3 (0.1%) | - |
| Grandmother | 55 (0.6%) | 13 (1.4%) | 20 (0.7%) | 3 (0.8%) | 19 (0.3%) | - |
| Stepfather | 11 (0.1%) | 1 (0.1%) | 5 (0.2%) | 2 (0.5%) | 3 (0.1%) | - |
| Stepmother | 39 (0.4%) | 7 (0.8%) | 15 (0.5%) | 3 (0.8%) | 13 (0.2%) | - |
| Missing/Other | 251 (2.6%) | 134 (14.4%) | 56 (1.8%) | 60 (15.9%) | 0 (0.0%) | - |
| Employment status |  |  |  |  |  |  |
| Education | 215 (2.2%) | 38 (4.1%) | 83 (2.7%) | 12 (3.2%) | 87 (1.6%) | 0.1%^a^ |
| Homemaker/full-time parent | 1107 (11.3%) | 68 (7.3%) | 354 (11.7%) | 38 (10.1%) | 662 (11.9%) | 0.3%^a^ |
| Self employed | 875 (8.9%) | 84 (9.0%) | 269 (8.9%) | 28 (7.4%) | 497 (8.9%) | 14.1%^a^ |
| Unemployed/Other | 403 (4.1%) | 32 (3.4%) | 155 (5.1%) | 23 (6.1%) | 201 (3.6%) | 4.1%^a^ |
| Working full time | 3480 (35.5%) | 287 (30.8%) | 1153 (38.0%) | 132 (34.9%) | 1950 (35.0%) | 61.2%^a^ |
| Working part time | 3483 (35.5%) | 227 (24.4%) | 993 (32.8%) | 123 (32.5%) | 2179 (39.1%) | 20.2%^a^ |
| Missing | 242 (2.5%) | 195 (20.9%) | 25 (0.8%) | 22 (5.8%) | 0 (0.0%) | - |
| Country |  |  |  |  |  |  |
| England | 8609 (97.8%) | 779 (83.7%) | 2630 (86.7%) | 322 (85.2%) | 4975 (89.2%) | 84.3%^b^ |
| Northern Ireland | 127 (1.3%) | 19 (2.0%) | 39 (1.3%) | 6 (1.6%) | 65 (1.2%) | 2.8%^b^ |
| Scotland | 664 (6.8%) | 67 (7.2%) | 227 (7.5%) | 29 (7.7%) | 349 (6.3%) | 8.2%^b^ |
| Wales | 360 (3.7%) | 36 (3.9%) | 126 (4.2%) | 18 (4.8 %) | 183 (3.3%) | 4.7%^b^ |
| Missing | 45 (0.5%) | 30 (3.2%) | 10 (0.3%) | 3 (0.8%) | 4 (0.1%) | - |
| Parent/carer ethnicity |  |  |  |  |  |  |
| White: British, Irish, other | 8856 (90.3%) | 654 (70.2%) | 2730 (90.0%) | 329 (87.0%) | 5246 (94.1%) | 81.7%^c^ |
| Other ethnic backgrounds | 562 (5.7%) | 111 (11.9%) | 210 (6.9%) | 21 (5.6%) | 223 (4.0%) | 18.3% ^c^ |
| Missing | 387 (3.9%) | 166 (17.8%) | 92 (3.0%) | 28 (7.4%) | 107 (1.9%) | - |
| Parent/carer gender |  |  |  |  |  |  |
| Female | 8985 (91.6%) | 796 (85.5%) | 2759 (91.0%) | 307 (81.2%) | 5229 (93.8%) | 50.6%^d^ |
| Male | 717 (7.3%) | 90 (9.7%) | 247 (8.1%) | 39 (10.3%) | 347 (6.2%) | 49.4%^d^ |
| Other/Unknown | 103 (1.1%) | 45 (4.8%) | 26 (0.9%) | 32 (8.5%) | 0 (0%) | - |
| Household income |  |  |  |  |  |  |
| < £16,000 p.a. | 902 (9.2%) | 78 (8.4%) | 367 (12.1%) | 60 (15.9%) | 414 (7.4%) | 12.8%^e^ |
| ≥ £16,000 p.a. | 8009 (81.7%) | 585 (62.8%) | 2443 (80.6%) | 270 (71.4%) | 4802 (86.1%) | 87.2%^e^ |
| Missing | 984 (9.1%) | 268 (28.8%) | 222 (7.3%) | 48 (12.7%) | 360 (6.5%) | - |

Notes. ^a^ Proportion of economically active UK population in Mar-May 2020 (Office for National Statistics, 2022a).

^b^ 2020 UK population by region (Clark., 2022).

^c^ Census 2021 data by ethnic group (Office for National Statistics, 2022b).

^d^ Census 2011 data by gender (GOV.UK, 2018).

^e^ Proportion of UK households in 2020 with an average household income < £16,000 p.a. (Office for National Statistics, 2021).

Table S2

Predictor variables (at Baseline) of the final sample in comparison to the excluded participants and national figures

|  | Initial sample | Attrition | | | Final sample | National figures |
| --- | --- | --- | --- | --- | --- | --- |
|  |  | Missing outcomes | Single participation | Missing predictors |  |  |
|  | (*N*=9805) | (*N*=931) | (*N*=5576) | (*N*=378) | (*N*=5576) |  |
| Parental age |  |  |  |  |  |  |
| Mean (SD) | 40.5 (6.72) | 38.2 (7.09) | 39.6 (6.94) | 41.4 (6.93) | 41.2 (6.43) | - |
| Missing | 644 (6.6%) | 288 (30.9%) | 179 (5.9%) | 177 (46.8%) | 0 (0.0%) | - |
| Parent education |  |  |  |  |  |  |
| Further | 1375 (14.0%) | 134 (14.4%) | 503 (16.6%) | 66 (17.5%) | 693 (12.4%) | 11.6%^a^ |
| Higher | 7336 (74.8%) | 508 (54.6%) | 2152 (71.0%) | 239 (63.2%) | 4513 (80.9%) | 50.1%^a^ |
| Secondary or below | 852 (8.7%) | 94 (10.1%) | 352 (11.6%) | 51 (13.5%) | 370 (6.6%) | 38.3%^a^ |
| Missing | 242 (2.5%) | 195 (20.9%) | 25 (0.8%) | 22 (5.8%) | 0 (0.0%) | - |
| Single Adult Household |  |  |  |  |  |  |
| Non single adult | 7976 (81.3%) | 618 (66.4%) | 2418 (79.7%) | 215 (56.9%) | 4811 (86.3%) | 85.3%^b^ |
| Single adult | 1445 (14.7%) | 103 (11.1%) | 524 (17.3%) | 80 (21.2%) | 765 (13.7%) | 14.7% ^b^ |
| Missing | 384 (3.9%) | 210 (22.6%) | 90 (3.0%) | 83 (22.0%) | 0 (0.0%) | - |
| Mental Health |  |  |  |  |  |  |
| Depression/Anxiety/Other | 1696 (17.3%) | 101 (10.8%) | 589 (19.4%) | 86 (22.8%) | 950 (17.0%) | 17%^c^ |
| None | 8109 (82.7%) | 830 (89.2%) | 2443 (80.6%) | 292 (77.2%) | 4626 (83.0%) | 83%^c^ |
| Work at home |  |  |  |  |  |  |
| Not worked | 3033 (30.9%) | 57 (6.1%) | 1153 (38.0%) | 133 (35.2%) | 1800 (32.3%) | - |
| Worked at home | 4293 (43.8%) | 58 (6.2%) | 1330 (43.9%) | 152 (40.2%) | 2885 (51.7%) | - |
| Worked out of the home | 1486 (15.2%) | 20 (2.1%) | 547 (18.0%) | 91 (24.1%) | 891 (16%) | - |
| Missing | 993 (10.1%) | 796 (85.5%) | 2 (0.1%) | 2 (0.5%) | 0 (0.0%) | - |
| Children's age |  |  |  |  |  |  |
| <4 | 1421 (14.5%) | 272 (29.2%) | 463 (15.3%) | 28 (7.4%) | 672 (12.1%) | 21.0%^d^ |
| 4-10 | 3086 (31.5%) | 226 (24.3%) | 893 (29.5%) | 108 (28.6%) | 1887 (33.8%) | 40.7%^d^ |
| 11-17 | 1939 (19.8%) | 102 (11.0%) | 606 (20.0%) | 118 (31.2%) | 1147 (20.6%) | 38.3%^d^ |
| Mixed ages | 3215 (32.8%) | 221 (23.7%) | 1050 (34.6%) | 110 (29.1%) | 1870 (33.5%) | - |
| Missing | 144 (1.5%) | 110 (11.8%) | 20 (0.7%) | 14 (3.7%) | 0 (0.0%) | - |
| Child SEN/ND Status |  |  |  |  |  |  |
| No SEN/ND | 8206 (83.7%) | 723 (77.7%) | 2539 (83.7%) | 227 (60.1%) | 4797 (86.0%) | 84.1%^e^ |
| SEN/ND | 1345 (13.7%) | 90 (9.7%) | 434 (14.3%) | 74 (19.6%) | 779 (14.0%) | 15.9%^e^ |
| Missing | 254 (2.6%) | 118 (12.7%) | 59 (1.9%) | 77 (20.4%) | 0 (0.0%) | - |

Notes. ^a^ UK educational attainment in 2021(O.E.C.D. Statistics, n.d.)

^b^ Proportion of lone parent households in 2019 (Office for National Statistics, 2021b)

^c^ Proportion of surveyed adults in England meeting the criteria for a common mental disorder in 2014 (NHS Digital, 2016).

^d^ UK mid-2020 population estimates based on the single year of age (Office for National Statistics, 2021c)

^e^ The percentage of pupils with an Education, Health and Care (EHC) plan or SEN support plan in Academic Year 2020/21(GOV.UK, n.d.).

**Figure S1**

*Frequency of participants’ first (Baseline) survey per month*


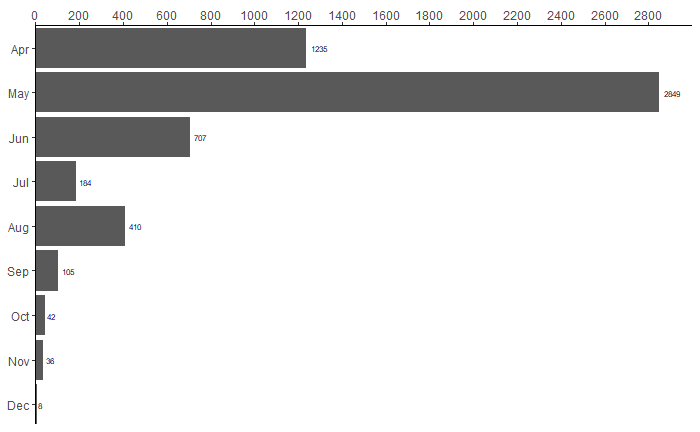


Measures: Personal and Family Characteristics

- Parental age – Participants reported the age in years for all household members (e.g., mother, father, brother and etc.), their own gender (‘Female’, ‘Male’, ‘Other/Prefer not to say’), and their relationship to a child (e.g., parent, grandparent, step-parent, or other) when completing their first survey for the study. This information was used to determine the age of the participant. Due to the information available, it was not possible to retrieve age information for the participants, who identified their gender as ‘Other/Prefer not to say’ and thus these participants will have to be excluded from the analyses. This variable was grand mean centered and used as a continuous predictor during mixed-effect modelling. For the secondary analysis, it was dichotomized as ‘younger than average’ or ‘older than average’.
- Parental Education – Participants were asked a multiple-choice question ‘What is your highest level of educational attainment?’ with 6 possible answers (‘No qualifications’, ‘Completed GCSE/CSE/O-levels or equivalent (at school till aged 16)’, ‘Completed post-16 vocational course’, ‘A-levels or equivalent (at school till aged 18)’, ‘Undergraduate degree or professional qualification’, or ‘Postgraduate degree’). These responses were combined to devise 3 different education levels: ‘Secondary or below’ (‘No qualifications’ or ‘Completed GCSE/CSE/O-levels or equivalent (at school till aged 16)’) ‘Further’ (‘Completed post-16 vocational course’ or ‘A-levels or equivalent (at school till aged 18)’) and ‘Higher’ (‘Undergraduate degree or professional qualification’ or ‘Postgraduate degree’). ‘Higher’ group was used as a reference category for group comparisons.
- Single Adult Household – Parents/carers were asked to list the ages of all other members of their household (in years). Using this information, a single-adult household was defined as a household in which the parent/carer taking the survey was the only adult over the age of 18 years. Any negative (impossible) values produced by this estimation were treated as missing and thus excluded from analyses. Single-adult households were coded as a dichotomous variable (‘Non single adult’ or ‘Single adult’).
- Parent Mental Health Diagnosis – When asked ‘Does anyone in your household have any of the following medical conditions?’ participants were also asked to report on their own conditions including ‘Clinically-diagnosed depression’, ‘Clinically-diagnosed anxiety’, and/or ‘Another clinically-diagnosed mental health condition’. This information was used to create a dichotomous indicator for whether the participant has a pre-existing mental health diagnosis (‘Depression/Anxiety/Other’ or ‘No’).
- Work at home – During each survey, participants were asked ‘Over the last week, have you worked?’ (‘Not at all’, ‘Part-time’, or ‘Full-time’) and ‘Has your work been at home or out of the home?’ (‘At home’, ‘Out of the home’, ‘Both’). As the ‘Both’ option in the latter question has only been added to the Co-SPACE (but not Co-SPYCE) questionnaire on 27th of October 2020, it was unlikely to produce enough meaningful data for a category of its own. Given it also represents the challenges of working at home, any ‘Both’ responses will be counted towards ‘At home’ category. Missing responses for this variable were imputed by taking a response from a previous questionnaire when available. If no answers were recorded at any time points (fully missing), participant were excluded from analyses. For the primary analysis using mixed-effect modelling, answers to these questions were used to devise a categorical time-variant predictor indicating whether the participant has worked at home over the last week: ‘Worked at home’, ‘Worked out of the home’, or ‘Not-worked’. ‘Worked at home’ was used as a reference category. For the secondary analysis, this information was recoded as time-invariant categorical predictor including potential change in working conditions over time: ‘worked at home’, ‘worked out of home’, ‘worked at home and out of home’, ‘not worked’, and ‘worked and not’.
- Children’s age – During the first survey participants were also asked to report the age in years for each child in the household. This information was used to determine 4 age groups for the children in the household: ‘Pre-schoolers only (<4)’, ‘Primary school aged only (4-10)’, ‘Secondary school aged only (11-17)’, or ‘Mixed/other’. ‘Primary school aged only (4-10)’ was used a reference category for planned comparisons in mixed-effect modelling, but ‘Pre-school only (<4)’ was used as a reference category during the secondary analysis.
- Child’s SEN/ND status – During their completion of the first survey, participants were asked whether the child they were reporting on had special education needs and whether any of the children in their household had Attention-deficit disorder (ADD)/Attention deficit hyperactivity disorder (ADHD) or Autism spectrum disorder (ASD). These indicators were used to devise a dichotomous between-subject variable for whether they had a child with special education needs/ neurodevelopmental difference (SEN/ND): ‘SEN/ND’ or ‘No SEN/ND’.

Model Selection Process for Mixed-effect Modelling with Growth Curve Analysis

Before the main analysis was conducted, we evaluated which fixed effects should be included in each final model. Growth curve modelling of ‘Time’ (i.e., the month when the survey was completed) was conducted to examine whether linear, quadratic, or cubic trends should be modelled. Each predictor variable was then assessed (one at a time) to determine whether they improved the model as a main effect and via an interaction with included ‘Time’ trends. The model indices, including Akaike Information Criteria (AIC), Bayesian Information Criterion (BIC), and χ2 log-likelihood change, were used to compare model fit. Given that BIC tends to heavily penalize model fit for increased parameters (Kuha, 2004), AIC and χ2 test were prioritized.

Table S3

Model selection and fit indices for parental anxiety

|  |  | *df* | *AIC* | *BIC* | *Chi2* | *p* |
| --- | --- | --- | --- | --- | --- | --- |
| Model building | |  |  |  |  |  |
| **M0** | **intercept only** | **3** | **139028** | **139053** |  |  |
| **M1** | **M0 + linear time** | **4** | **138956** | **138988** | **74.81** | **<.001** |
| **M2** | **M1 + quadratic time** | **5** | **138903** | **138944** | **54.40** | **<.001** |
| M3 | M2 + cubic time | 6 | 138902 | 138950 | 3.30 | .069 |
| M2 + Parental age | |  |  |  |  |  |
| **M4** | **M2 + main effect** | **6** | **138854** | **138902** | **51.56** | **<.001** |
| **M5** | **M4 + linear interaction** | **7** | **138854** | **138911** | **1.36** | **.243** |
| **M6** | **M5 + quadratic interaction** | **8** | **138849** | **138914** | **7.17** | **.007** |
| M2 + Parental education | |  |  |  |  |  |
| **M7** | **M2 + main effect** | **7** | **138801** | **138858** | **106.23** | **<.001** |
| **M8** | **M7 + linear interaction** | **9** | **138799** | **138872** | **5.89** | **.053** |
| **M9** | **M8 + quadratic interaction** | **11** | **138795** | **138884** | **8.17** | **.017** |
| M2 + Single adult | |  |  |  |  |  |
| **M10** | **M2 + main effect** | **6** | **138775** | **138823** | **130.36** | **<.001** |
| M11 | M10 + linear interaction | 7 | 138774 | 138831 | 2.43 | .119 |
| M12 | M11 + quadratic interaction | 8 | 138776 | 138841 | 0.38 | .539 |
| M2 + Parental MH | |  |  |  |  |  |
| **M13** | **M2 + main effect** | **6** | **137951** | **138000** | **953.90** | **<.001** |
| M14 | M13 + linear interaction | 7 | 137953 | 138009 | 0.48 | .488 |
| M15 | M14 + quadratic interaction | 8 | 137955 | 138019 | 0.13 | .714 |
| M2 + Work | |  |  |  |  |  |
| **M16** | **M2 + main effect** | **7** | **138899** | **138956** | **7.98** | **.018** |
| **M17** | **M16 + linear interaction** | **9** | **138893** | **138966** | **10.12** | **.006** |
| **M18** | **M17 + quadratic interaction** | **11** | **138890** | **138979** | **6.83** | **.033** |
| M2 + Children’s age group | |  |  |  |  |  |
| **M19** | **M2 + main effect** | **8** | **138893** | **138957** | **16.51** | **<.001** |
| **M20** | **M19 + linear interaction** | **11** | **138886** | **138975** | **12.29** | **.006** |
| **M21** | **M20 + quadratic interaction** | **14** | **138868** | **138981** | **24.75** | **<.001** |
| M2 + Child SEN/ND | |  |  |  |  |  |
| **M22** | **M2 + main effect** | **6** | **138798** | **138846** | **107.28** | **<.001** |
| **M23** | **M22 + linear interaction** | **7** | **138775** | **138831** | **25.12** | **<.001** |
| M24 | M23 + quadratic interaction | 8 | 138777 | 138841 | 0.02 | .876 |
| FINAL MODEL: all identified effects included | | | | | | |
|  |  | 33 | 137682 | 137949 |  |  |

*Note.* Final model of the main analysis includes all effects identified as improving model fit (in bold) in the previous steps of the main analysis.

Table S4

Model selection and fit indices for parental stress

|  |  | *df* | *AIC* | *BIC* | *Chi2* | *p* |
| --- | --- | --- | --- | --- | --- | --- |
| Model building | |  |  |  |  |  |
| **M0** | **intercept only** | **3** | **157121** | **157145** |  |  |
| **M1** | **M0 + linear time** | **4** | **157119** | **157152** | **3.70** | **.054** |
| **M2** | **M1 + quadratic time** | **5** | **156752** | **156792** | **369.43** | **<.001** |
| **M3** | **M2 + cubic time** | **6** | **156689** | **156738** | **64.52** | **<.001** |
| M3 + Parental age | |  |  |  |  |  |
| **M4** | **M3 + main effect** | **7** | **156628** | **156685** | **63.06** | **<.001** |
| **M5** | **M4 + linear interaction** | **8** | **156630** | **156694** | **0.77** | **.380** |
| **M6** | **M5 + quadratic interaction** | **9** | **156620** | **156692** | **11.90** | **<.001** |
| M7 | M6 + cubic interaction | 10 | 156622 | 156702 | 0.13 | .716 |
| M3 + Parental education | |  |  |  |  |  |
| **M8** | **M3 + main effect** | **8** | **156688** | **156753** | **5.4809** | **.065** |
| **M9** | **M8 + linear interaction** | **10** | **156690** | **156771** | **1.566** | **.457** |
| **M10** | **M9 + quadratic interaction** | **12** | **156681** | **156778** | **13.7202** | **.001** |
| **M11** | **M10 + cubic interaction** | **14** | **156677** | **156790** | **7.8827** | **.019** |
| M3 + Single adult | |  |  |  |  |  |
| **M12** | **M3 + main effect** | **7** | **156668** | **156724** | **23.65** | **<.001** |
| **M13** | **M12 + linear interaction** | **8** | **156670** | **156734** | **0.23** | **.630** |
| **M14** | **M13 + quadratic interaction** | **9** | **156664** | **156737** | **7.62** | **.006** |
| M15 | M14 + cubic interaction | 10 | 156666 | 156747 | 0.18 | .671 |
| M3 + Parental MH | |  |  |  |  |  |
| **M16** | **M3 + main effect** | **7** | **156037** | **156094** | **654.16** | **<.001** |
| M17 | M16 + linear interaction | 8 | 156039 | 156104 | 0.06 | .802 |
| M18 | M17 + quadratic interaction | 9 | 156040 | 156113 | 1.36 | .244 |
| M19 | M18 + cubic interaction | 10 | 156039 | 156120 | 3.16 | .076 |
| M3 + Work | |  |  |  |  |  |
| **M20** | **M3 + main effect** | **8** | **156674** | **156739** | **19.50** | **<.001** |
| **M21** | **M20 + linear interaction** | **10** | **156659** | **156740** | **18.84** | **<.001** |
| **M22** | **M21 + quadratic interaction** | **12** | **156649** | **156746** | **13.90** | **.001** |
| **M23** | **M22 + cubic interaction** | **14** | **156638** | **156751** | **15.35** | **<.001** |
| M3 + Children’s age group | |  |  |  |  |  |
| **M24** | **M3 + main effect** | **9** | **156653** | **156726** | **42.02** | **<.001** |
| **M25** | **M24 + linear interaction** | **12** | **156656** | **156753** | **3.33** | **.344** |
| **M26** | **M25 + quadratic interaction** | **15** | **156620** | **156741** | **42.00** | **<.001** |
| **M27** | **M26 + cubic interaction** | **18** | **156614** | **156759** | **12.57** | **.006** |
| M3 + Child SEN/ND | |  |  |  |  |  |
| **M28** | **M3 + main effect** | **7** | **156605** | **156662** | **86.06** | **<.001** |
| **M29** | **M28 + linear interaction** | **8** | **156600** | **156664** | **7.73** | **.005** |
| **M30** | **M29 + quadratic interaction** | **9** | **156597** | **156669** | **5.07** | **.024** |
| M31 | M30 + cubic interaction | 10 | 156599 | 156679 | 0.07 | .793 |
| FINAL MODEL: all identified effects included | | | | | | |
|  |  | 44 | 155794 | 156149 |  |  |

*Note.* Final model of the main analysis includes all effects identified as improving model fit (in bold) in the previous steps of the main analysis.

Table S5

Model selection and fit indices for parental depression

|  |  | *df* | *AIC* | *BIC* | *Chi2* | *p* |
| --- | --- | --- | --- | --- | --- | --- |
| Model building | |  |  |  |  |  |
| **M0** | **intercept only** | **3** | **157573** | **157597** |  |  |
| **M1** | **M0 + linear time** | **4** | **157508** | **157541** | **66.53** | **<.001** |
| **M2** | **M1 + quadratic time** | **5** | **157199** | **157240** | **311.26** | **<.001** |
| **M3** | **M2 + cubic time** | **6** | **157131** | **157180** | **70.06** | **<.001** |
| M3 + Parental age | |  |  |  |  |  |
| **M4** | **M3 + main effect** | **7** | **157131** | **157188** | **1.86** | **.173** |
| **M5** | **M4 + linear interaction** | **8** | **157133** | **157198** | **0.25** | **.619** |
| **M6** | **M5 + quadratic interaction** | **9** | **157120** | **157193** | **14.94** | **<.001** |
| M7 | M6 + cubic time | 10 | 157121 | 157202 | 1.33 | .249 |
| M3 + Parental education | |  |  |  |  |  |
| **M8** | **M3 + main effect** | **8** | **157065** | **157130** | **70.07** | **<.001** |
| M9 | M8 + linear interaction | 10 | 157068 | 157149 | 1.20 | .550 |
| M10 | M9 + quadratic interaction | 12 | 157067 | 157164 | 5.27 | .072 |
| M11 | M10 + cubic time | 14 | 157067 | 157180 | 4.08 | .130 |
| M3 + Single adult | |  |  |  |  |  |
| **M12** | **M3 + main effect** | **7** | **156952** | **157008** | **181.34** | **<.001** |
| **M13** | **M12+ linear interaction** | **8** | **156953** | **157018** | **0.89** | **.346** |
| **M14** | **M13 + quadratic interaction** | **9** | **156947** | **157019** | **8.27** | **.004** |
| M15 | M14 + cubic time | 10 | 156949 | 157029 | 0.04 | .850 |
| M3 + Parental MH | |  |  |  |  |  |
| **M16** | **M3 + main effect** | **7** | **156390** | **156447** | **742.73** | **<.001** |
| **M17** | **M16 + linear interaction** | **8** | **156390** | **156455** | **2.13** | **.145** |
| **M18** | **M17 + quadratic interaction** | **9** | **156392** | **156465** | **0.01** | **.994** |
| **M19** | **M18 + cubic time** | **10** | **156390** | **156471** | **4.42** | **.036** |
| M3 + Work | |  |  |  |  |  |
| **M20** | **M3 + main effect** | **8** | **157067** | **157131** | **68.41** | **<.001** |
| **M21** | **M20 + linear interaction** | **10** | **157059** | **157140** | **11.53** | **.003** |
| M22 | M21 + quadratic interaction | 12 | 157062 | 157159 | 0.84 | .656 |
| M23 | M22 + cubic time | 14 | 157062 | 157175 | 4.12 | .127 |
| M3 + Children’s age group | |  |  |  |  |  |
| **M24** | **M3 + main effect** | **9** | **157109** | **157181** | **28.48** | **<.001** |
| **M25** | **M24 + linear interaction** | **12** | **157106** | **157203** | **8.32** | **.040** |
| **M26** | **M25 + quadratic interaction** | **15** | **157096** | **157217** | **16.80** | **.001** |
| M27 | M26 + cubic time | 18 | 157097 | 157243 | 4.10 | .251 |
| M3 + Child SEN/ND | |  |  |  |  |  |
| **M28** | **M3 + main effect** | **7** | **157008** | **157065** | **125.24** | **<.001** |
| **M29** | **M28 + linear interaction** | **8** | **157007** | **157072** | **2.59** | **.107** |
| **M30** | **M29 + quadratic interaction** | **9** | **157005** | **157078** | **4.51** | **.034** |
| **M31** | **M30 + cubic time** | **10** | **157004** | **157085** | **2.69** | **.101** |
| FINAL MODEL: all identified effects included | | | | | | |
|  |  | 31 | 156127 | 156377 |  |  |

*Note.* Final model of the main analysis includes all effects identified as improving model fit (in bold) in the previous steps of the main analysis.

Model Selection Process for Latent Class Growth Mixture Modelling (LCGMM)

Models with two to five latent classes were fitted for each outcome variable separately. The functional form for each model was determined based on the outcome of the growth curve modelling in mixed effect modelling. Due to negative residual variances and correlations greater than 1 between the latent variables, the linear, quadratic, and cubic slopes were constrained to 0, when included (resulting in Latent Class Growth Analysis model specifications). The best fitting number of classes was determined based on BIC and the Vuong-Lo-Mendell-Rubin adjusted likelihood ratio (VLMR- LRT; Lo, Mendell, & Rubin, 2001; Vuong, 1989) test. Diagnostic criteria such as entropy index and smallest class size were also examined (see Table S8).

Table S6

Model fit statistics and diagnostic criteria for 2 to 5 class models per DASS sub-scale

|  | BIC | VLMR- LRT | | Entropy | Smallest class |
| --- | --- | --- | --- | --- | --- |
|  |  | -2LL | *p* |  |  |
| Anxiety |  |  |  |  |  |
| 2 Class | 136426.02 | 2367.34 | <.001 | .935 | 10% |
| **3 Class** | **135369.17** | **1091.36** | **.016** | **.930** | **3%** |
| 4 Class | 134490.52 | 913.16 | .521 | .916 | 2% |
| 5 Class | 133862.09 | 662.93 | .646 | .909 | 2% |
| Stress |  |  |  |  |  |
| 2 Class | 156124.16 | 620.18 | <.001 | .751 | 13% |
| 3 Class | 155651.39 | 515.90 | .446 | .747 | 9% |
| 4 Class | 155366.93 | 327.60 | .004 | .746 | 7% |
| **5 Class** | **155244.30** | **165.76** | **.038** | **.727** | **5%** |
| Depression |  |  |  |  |  |
| 2 Class | 155466.27 | 1738.09 | <.001 | .881 | 14% |
| 3 Class | 154699.93 | 809.48 | .074 | .862 | 8% |
| 4 Class | 154136.99 | 606.07 | .059 | .854 | 5% |
| **5 Class** | **153703.48** | **476.64** | **.041** | **.842** | **4%** |

*Note*. Bold text indicates model met fit criteria. BIC = Bayesian information criterion; -2LL = 2 times the log-likelihood difference; VLMR-LRT = Vuong-Lo-Mendell- Rubin adjusted likelihood ratio test. Entropy

Table S7

Means and standard deviations of average DASS scores across time, per subscale, and categorical predictors

|  |  | Anxiety | |  | Stress | |  | Depression | |
| --- | --- | --- | --- | --- | --- | --- | --- | --- | --- |
|  |  | *M* | *SD* |  | *M* | *SD* |  | *M* | *SD* |
| Total sample |  | 4.29 | 6.18 |  | 12.36 | 8.96 |  | 9.01 | 9.21 |
| Education |  |  |  |  |  |  |  |  |  |
| Further |  | 6.04 | 7.73 |  | 13.27 | 9.72 |  | 11.29 | 10.65 |
| Higher |  | 3.95 | 5.75 |  | 12.29 | 8.76 |  | 8.60 | 8.80 |
| Secondary or below |  | 5.66 | 7.77 |  | 11.67 | 10.08 |  | 10.41 | 10.92 |
| Single Adult Household |  |  |  |  |  |  |  |  |  |
| Non single adult |  | 3.98 | 5.85 |  | 12.17 | 8.77 |  | 8.46 | 8.70 |
| Single adult |  | 6.34 | 7.76 |  | 13.63 | 10.04 |  | 12.66 | 11.37 |
| Parent Mental Health Diagnosis |  |  |  |  |  |  |  |  |  |
| Depression/Anxiety/Other |  | 9.14 | 8.67 |  | 18.24 | 9.93 |  | 15.46 | 11.44 |
| No |  | 3.33 | 5.03 |  | 11.20 | 8.27 |  | 7.73 | 8.11 |
| Parent work |  |  |  |  |  |  |  |  |  |
| Not-working |  | 5.10 | 7.04 |  | 12.76 | 9.50 |  | 10.61 | 10.39 |
| Working at home |  | 3.82 | 5.58 |  | 12.44 | 8.68 |  | 8.40 | 8.45 |
| Working out of home |  | 4.22 | 6.08 |  | 11.64 | 8.76 |  | 8.17 | 8.80 |
| Children's age |  |  |  |  |  |  |  |  |  |
| Pre-schoolers (< 4 years) only |  | 3.56 | 5.21 |  | 12.06 | 8.23 |  | 7.92 | 8.16 |
| Children (4-10 years) only |  | 4.26 | 6.06 |  | 12.64 | 8.98 |  | 9.01 | 9.07 |
| Adolescents (11-17 years) only |  | 4.62 | 6.73 |  | 11.18 | 9.00 |  | 10.06 | 10.03 |
| Mixed ages |  | 4.33 | 6.20 |  | 12.90 | 9.05 |  | 8.69 | 9.05 |
| Child SEN/ND Status |  |  |  |  |  |  |  |  |  |
| No SEN/ND |  | 3.97 | 5.82 |  | 11.95 | 8.73 |  | 8.50 | 8.78 |
| SEN/ND |  | 6.19 | 7.75 |  | 14.80 | 9.87 |  | 12.05 | 10.94 |

*Note*. Category of ‘Mixed ages’ refers to families that had children falling into multiple age categories (e.g., pre-schoolers and adolescents). SEN/ND refers to the child’s special education needs or neurodevelopmental disorders.

Table S8

Correlation matrix for continuous variables

|  | Parent Age | Parent Anxiety | Parent Stress |
| --- | --- | --- | --- |
| Parent Anxiety | -.09** |  |  |
| Parent Stress | -.10** | .68** |  |
| Parent Depression | -.01* | .63** | .71** |

* *p* < .05, ** *p* < .001

Summary of Main Effects in Growth Curve Mixed-effect Models

Being a single adult in the household, having a previous mental health diagnosis, or having a child with SEN/ND were all significantly associated with higher anxiety, stress, and depression symptoms. Parents’ age was negatively associated with all (anxiety, stress, and depression) symptoms. Completion of higher education was also associated with lower anxiety (compared to further or ‘secondary or lower’ education) and depression symptoms (compared to further education), but higher stress symptoms (compared to ‘secondary or lower’ education). Working out of the home was associated with lower stress and depression symptoms than working in the home. Yet, those who were not working reported lower stress and higher depression symptoms than those who were working at home. Having a child in the pre-school (<4 years) age range only was also associated with lower anxiety, stress, and depression when compared to having children in the 4–10-year-old age range. Having a child in the 11–16-year-old age range predicted lower stress than having a 4–10-year-olds, however, was associated with higher anxiety and depression.

Table S9

Estimates for final LCGMM models

| Classes | Intercept |  | Linear slope |  | Quadratic slope |  | Cubic slope |
| --- | --- | --- | --- | --- | --- | --- | --- |
|  | *Est (SE)* |  | *Est (SE)* |  | *Est (SE)* |  | *Est (SE)* |
| Anxiety |  |  |  |  |  |  |  |
| Low ^R^ | 2.67 (0.10) |  | -0.27 (0.04)** |  | 0.03 (<0.01)** |  |  |
| Consistently moderate | 11.49 (0.51) |  | 0.07 (0.25) |  | 0.02 (0.03) |  |  |
| High increasing | 21.26 (1.45) |  | 1.91 (0.75)* |  | -0.17 (0.09) |  |  |
| Stress |  |  |  |  |  |  |  |
| Low ^R^ | 10.50 (0.21) |  | -0.28 (0.15) |  | -0.08 (0.04) |  | 0.01 (<0.01)** |
| High to medium | 30.29 (1.16) |  | -2.74 (1.43) |  | -0.31 (0.33) |  | 0.05 (0.02)* |
| Low to high | 12.86 (2.04) |  | 3.44 (1.94) |  | -1.07 (0.47)* |  | 0.10 (0.03)* |
| Medium to consistently high | 19.94 (2.11) |  | 6.57 (1.13)** |  | -1.40 (0.31)** |  | 0.09 (0.02)** |
| Down and up | 23.43 (2.30) |  | -9.96 (2.12)** |  | 3.07 (0.56)** |  | -0.22 (0.04)** |
| Depression |  |  |  |  |  |  |  |
| Low ^R^ | 6.18 (0.20) |  | -0.07 (0.13) |  | -0.12 (0.03)** |  | 0.02 (<0.01)** |
| High to low | 28.10 (2.28) |  | -4.00 (2.73) |  | -0.16 (0.65) |  | 0.05 (0.04) |
| Low to medium | 10.16 (1.16) |  | 8.86 (1.28)** |  | -1.88 (0.44)** |  | 0.11 (0.04)* |
| Medium to high | 16.04 (1.21) |  | -4.41 (0.99)** |  | 1.34 (0.27)** |  | -0.08 (0.02)** |
| Consistently high | 31.78 (1.20) |  | 0.34 (1.02) |  | -0.10 (0.27) |  | 0.01 (0.02) |

*Notes*. ^R^ Reference groups; * *p*<.05; ** *p*<.001

Table S10

Relative odds ratios and 95% confidence intervals from multinomial logistic regression comparing characteristics of latent class membership for anxiety

| Characteristic | Low | Consistently moderate | High increasing |
| --- | --- | --- | --- |
| *N* (%) | 4573 (82%) | 846 (15%) | 157 (3%). |
| Parental age |  |  |  |
| Below average | Ref | **1.37 [1.15:1.65]** | **1.66 [1.13:2.44]** |
| Above average | Ref | Ref | Ref |
| Parent education |  |  |  |
| Higher | Ref | Ref | Ref |
| Further | Ref | **1.38 [1.11:1.73]** | **2.13 [1.41:3.22]** |
| Secondary or below | Ref | **1.37 [1.03:1.84]** | **2.00 [1.16:3.44]** |
| Single Adult Household | | | |
| No | Ref | Ref | Ref |
| Yes | Ref | **1.64 [1.33:2.02]** | **2.06 [1.41:3.02]** |
| Mental Health Diagnosis of Depression/Anxiety/Other | | | |
| No | Ref | Ref | Ref |
| Yes | Ref | **4.89 [4.11:5.81]** | **11.97 [8.40:17.05]** |
| Work |  |  |  |
| At home | Ref | Ref | Ref |
| Out of home | Ref | 1.09 [0.80:1.49] | 0.66 [0.33:1.35] |
| At home and not | Ref | 1.03 [0.75:1.41] | 0.98 [0.51:1.89] |
| Not worked | Ref | **1.51 [1.20:1.91]** | 1.25 [0.79:1.98] |
| Worked and not | Ref | **1.25 [1.01:1.54]** | 0.76 [0.48:1.21] |
| Children's age |  |  |  |
| < 4 | Ref | Ref | Ref |
| 4-10 | Ref | **1.54 [1.13:2.09]** | 1.71 [0.86:3.41] |
| 11-17 | Ref | **1.80 [1.27:2.55]** | **2.38[1.12:5.07]** |
| Mixed ages | Ref | **1.58 [1.17:2.14]** | 1.65 [0.83:3.27] |
| Child SEN/ND Status | | | |
| No | Ref | Ref | Ref |
| Yes | Ref | **1.62 [1.31:1.99]** | **1.65 [1.11:2.47]** |

Table S11

Membership in each latent class group for parental anxiety according to personal and family characteristics

| Characteristic | Low ^R^ | Consistently moderate | High increasing |
| --- | --- | --- | --- |
| *N* (%) | 4573 (82%) | 846 (15%)** | 157 (3%)** |
| Parental age |  |  |  |
| Below average | 2405 (52.6%) | 482 (57.0%)** | 95 (60.5%)* |
| Above average ^R^ | 2168 (47.4%) | 364 (43.0%) | 62 (39.5%) |
| Parent education |  |  |  |
| Higher ^R^ | 3806 (83.2%) | 611 (72.2%) | 96 (61.1%) |
| Further | 498 (10.9%) | 154 (18.2%)* | 41 (26.1%)** |
| Secondary or below | 269 (5.9%) | 81 (9.6%)* | 20 (12.7%)* |
| Single Adult Household | | | |
| No ^R^ | 4045 (88.5%) | 657 (77.7%) | 109 (69.4%) |
| Yes | 528 (11.5%) | 189 (22.3%)** | 48 (30.6%)** |
| Mental Health Diagnosis of Depression/Anxiety/Other | | | |
| No ^R^ | 4067 (88.9%) | 504 (59.6%) | 55 (35.0%) |
| Yes | 506 (11.1%) | 342 (40.4%)** | 102 (65.0%)** |
| Work |  |  |  |
| At home ^R^ | 1480 (32.4%) | 204 (24.1%) | 42 (26.8%) |
| Out of home | 421 (9.2%) | 73 (8.6%) | 11 (7.0%) |
| At home and not | 458 (10.0%) | 67 (7.9%) | 13 (8.3%) |
| Not worked | 781 (17.1%) | 227 (26.8%)** | 52 (33.1%) |
| Worked and not | 1433 (31.3%) | 275 (32.5%)* | 39 (24.8%) |
| Children's age |  |  |  |
| < 4 ^R^ | 590 (12.9%) | 70 (8.3%) | 12 (7.6%) |
| 4-10 | 1544 (33.8%) | 290 (34.3%)* | 53 (33.8%) |
| 11-17 | 911 (19.9%) | 195 (23.0%)** | 41 (26.1%)* |
| Mixed ages | 1528 (33.4%) | 291 (34.4%)* | 51 (32.5%) |
| Child SEN/ND Status | | | |
| No ^R^ | 4024 (88.0%) | 657 (77.7%) | 116 (73.9%) |
| Yes | 549 (12.0%) | 189 (22.3%)** | 41 (26.1%)* |

*Notes.* ^R^ Reference groups; * *p*<.05; ** *p*<.001

Table S12

Relative odds ratios and 95% confidence intervals from multinomial logistic regression comparing characteristics of latent class membership for stress

| Characteristic | Low | High to medium | Low to high | | Medium to consistently high | High and fluctuating |
| --- | --- | --- | --- | --- | --- | --- |
| *N* (%) | 4495 (81%) | 289 (5%) | | 228 (4%). | 394 (7%) | 170 (3%) |
| Parental age | | | | | | |
| Below average | Ref | 1.25 [0.94:1.67] | | 1.09 [0.79:1.51] | **1.41 [1.09:1.82]** | 1.45 [0.99:2.12] |
| Above average | Ref | Ref | | Ref | Ref | Ref |
| Parent education | | | | | | |
| Higher | Ref | Ref | | Ref | Ref | Ref |
| Further | Ref | 0.87 [0.58:1.30] | | 0.90 [0.57:1.42] | 1.00 [0.72:1.39] | 1.24 [0.79:1.94] |
| Secondary or below | Ref | 0.51 [0.26:1.02] | | 0.70 [0.36:1.35] | 1.11 [0.73:1.68] | 1.20 [0.66:2.17] |
| Single Adult Household | | | | | | |
| No | Ref | Ref | | Ref |  |  |
| Yes | Ref | 1.23 [0.86:1.77] | | 1.33 [0.90:1.98] | **1.52 [1.14:2.03]** | 1.13 [0.72:1.78] |
| Mental Health Diagnosis of Depression/Anxiety/Other | | | | | | |
| No | Ref | Ref | | Ref | Ref | Ref |
| Yes | Ref | **4.70 [3.57:6.20]** | | **2.04 [1.43:2.92]** | **5.21 [4.10:6.62]** | **3.54 [2.47:5.06]** |
| Work |  |  | |  |  |  |
| At home | Ref | Ref | | Ref | Ref | Ref |
| Out of home | Ref | **0.35 [0.18:0.69]** | | 0.67 [0.33:1.33] | 0.67 [0.41:1.10] | **1.88 [1.04:3.39]** |
| At home and not | Ref | 0.77 [0.47:1.24] | | 1.07 [0.61:1.88] | 1.17 [0.77:1.78] | 1.39 [0.72:2.69] |
| Not worked | Ref | 0.72 [0.49:1.05] | | 1.10 [0.69:1.74] | 1.22 [0.89:1.69] | 1.43 [0.85:2.39] |
| Worked and not | Ref | 0.86 [0.63:1.18] | | 1.41 [0.97:2.04] | 0.93 [0.69:1.26] | 1.50 [0.94:2.38] |
|  |  |  | |  |  |  |
| Children's age | | | | | | |
| < 4 | Ref | Ref | | Ref | Ref | Ref |
| 4-10 | Ref | 1.33 [0.86:2.05] | | **3.22 [1.58:6.54]** | 1.34 [0.88:2.03] | 1.15 [0.63:2.12] |
| 11-17 | Ref | **0.47 [0.26:0.86]** | | 1.95 [0.88:4.30] | 1.09 [0.67:1.78] | 1.11 [0.55:2.24] |
| Mixed ages | Ref | 1.08 [0.70:1.67] | | **2.25 [1.10:4.60]** | 1.34 [0.89:2.03] | 1.26 [0.70:2.27] |
| Child SEN/ND Status | | | | | | |
| No | Ref | Ref | | Ref | Ref | Ref |
| Yes | Ref | 1.41 [0.98:2.03] | | **1.79 [1.23:2.59]** | **1.81 [1.36:2.41]** | **1.93 [1.28:2.91]** |

Table S13

Membership in each latent class group for parental stress according to personal and family characteristics

| Characteristic | Low ^R^ | High to medium | Low to high | Medium to consistently high | High and fluctuating |
| --- | --- | --- | --- | --- | --- |
| *N* (%) | 4495 (81%) | **289 (5%)**** | **228 (4%)**** | **394 (7%)**** | **170 (3%)**** |
| Parental age | | | | |  |
| Below average | 2352 (52.3%) | 178 (61.6%) | 120 (52.6%) | **231 (58.6%)*** | 101 (59.4%) |
| Above average ^R^ | 2143 (47.7%) | 111 (38.4%) | 108 (47.4%) | 163 (41.4%) | 69 (40.6%) |
| Parent education | | | | |  |
| Higher ^R^ | 3659 (81.4%) | 240 (83.0%) | 187 (82.0%) | 300 (76.1%) | 127 (74.7%) |
| Further | 542 (12.1%) | 36 (12.5%) | 28 (12.3%) | 58 (14.7%) | 29 (17.1%) |
| Secondary or below | 294 (6.5%) | 13 (4.5%) | 13 (5.7%) | 36 (9.1%) | 14 (8.2%) |
| Single Adult Household | | | | |  |
| No ^R^ | 3939 (87.6%) | 241 (83.4%) | 183 (80.3%) | 308 (78.2%) | 140 (82.4%) |
| Yes | 556 (12.4%) | 48 (16.6%) | 45 (19.7%) | **86 (21.8%)*** | 30 (17.6%) |
| Mental Health Diagnosis of Depression/Anxiety/Other | | | | |  |
| No ^R^ | 3928 (87.4%) | 179 (61.9%) | 174 (76.3%) | 232 (58.9%) | 113 (66.5%) |
| Yes | 567 (12.6%) | **110 (38.1%)**** | **54 (23.7%)**** | **162 (41.1%)**** | **57 (33.5%)**** |
| Work |  |  |  |  |  |
| At home ^R^ | 1406 (31.3%) | 104 (36.0%) | 62 (27.2%) | 117 (29.7%) | 37 (21.8%) |
| Out of home | 428 (9.5%) | **12 (4.2%)*** | 16 (7.0%) | 26 (6.6%) | **23 (13.5%)*** |
| At home and not | 437 (9.7%) | 24 (8.3%) | 23 (10.1%) | 38 (9.6%) | 16 (9.4%) |
| Not worked | 820 (18.2%) | 54 (18.7%) | 39 (17.1%) | 108 (27.4%) | 39 (22.9%) |
| Worked and not | 1404 (31.2%) | 95 (32.9%) | 88 (38.6%) | 105 (26.6%) | 55 (32.4%) |
|  |  |  |  |  |  |
| Children's age | | | | |  |
| < 4 ^R^ | 563 (12.5%) | 36 (12.5%) | 14 (6.1%) | 42 (10.7%) | 17 (10.0%) |
| 4-10 | 1471 (32.7%) | 121 (41.9%) | **101 (44.3%)*** | 138 (35.0%) | 56 (32.9%) |
| 11-17 | 960 (21.4%) | **31 (10.7%)*** | 44 (19.3%) | 78 (19.8%) | 34 (20.0%) |
| Mixed ages | 1501 (33.4%) | 101 (34.9%) | **69 (30.3%)*** | 136 (34.5%) | 63 (37.1%) |
| Child SEN/ND Status | | | | |  |
| No ^R^ | 3941 (87.7%) | 241 (83.4%) | 181 (79.4%) | 302 (76.6%) | 132 (77.6%) |
| Yes | 554 (12.3%) | 48 (16.6%) | **47 (20.6%)*** | **92 (23.4%)**** | **38 (22.4%)*** |

*Notes.* ^R^ Reference groups; * *p*<.05; ** *p*<.001

Table S14

Relative odds ratios and 95% confidence intervals from multinomial logistic regression comparing characteristics of latent class membership for depression

| Characteristic | Low | High to low | Low to medium | Medium to high | Consistently high | |
| --- | --- | --- | --- | --- | --- | --- |
| *N* (%) | 4358 (78%) | 235 (4%) | 451 (8%). | 323 (6%) | 209 (4%) | |
| Parental age | | | | | |  |
| Below average | Ref | 1.21 [0.87:1.69] | 0.96 [0.76:1.23] | **1.32 [1.00:1.75]** | 1.12 [0.80:1.57] | |
| Above average | Ref | Ref | Ref | Ref | Ref | |
| Parent education | | | | | |  |
| Higher | Ref | Ref | Ref | Ref | Ref | |
| Further | Ref | 1.14 [0.75:1.74] | 1.06 [0.77:1.46] | **1.44 [1.03:2.01]** | **1.59 [1.10:2.32]** | |
| Secondary or below | Ref | 0.98 [0.53:1.78] | **1.52 [1.05:2.18]** | 1.16 [0.72:1.87] | 1.53 [0.95:2.48] | |
| Single Adult Household | | | | | |  |
| No | Ref | Ref | Ref |  |  | |
| Yes | Ref | 1.45 [0.99:2.14] | **1.73 [1.32:2.26]** | 1.16 [0.83:1.63] | **3.04 [2.20:4.21]** | |
| Mental Health Diagnosis of Depression/Anxiety/Other | | | | | |  |
| No | Ref | Ref | Ref | Ref | Ref | |
| Yes | Ref | **3.81 [2.79:5.22]** | **3.78 [2.99:4.78]** | **3.33 [2.54:4.37]** | **9.67 [7.10:13.17]** | |
| Work |  |  |  |  |  | |
| At home | Ref | Ref | Ref | Ref | Ref | |
| Out of home | Ref | **0.37 [0.17:0.84]** | 0.95 [0.62:1.46] | 1.06 [0.67:1.70] | 0.61 [0.29:1.32] | |
| At home and not | Ref | 1.09 [0.64:1.86] | 0.96 [0.63:1.48] | 1.02 [0.63:1.64] | 0.79 [0.37:1.65] | |
| Not worked | Ref | 1.19 [0.78:1.82] | **1.64 [1.21:2.23]** | 1.21 [0.83:1.75] | **2.60 [1.69:4.00]** | |
| Worked and not | Ref | 1.26 [0.88:1.81] | 1.10 [0.83:1.46] | 1.26 [0.92:1.73] | 1.52 [0.99:2.33] | |
|  |  |  |  |  |  | |
| Children's age | | | | | |  |
| < 4 | Ref | Ref | Ref | Ref | Ref | |
| 4-10 | Ref | 0.84 [0.52:1.35] | 1.20 [0.81:1.79] | **1.89 [1.16:3.09]** | 0.97 [0.54:1.72] | |
| 11-17 | Ref | 0.91 [0.52:1.61] | 1.39 [0.89:2.19] | **2.16 [1.25:3.76]** | 1.27 [0.68:2.40] | |
| Mixed ages | Ref | 0.73 [0.46:1.17] | 1.03 [0.69:1.54] | 1.34 [0.81:2.20] | 1.15 [0.66:2.01] | |
| Child SEN/ND Status | | | | | |  |
| No | Ref | Ref | Ref | Ref | Ref | |
| Yes | Ref | **1.55 [1.06:2.27]** | **1.57 [1.19:2.06]** | **1.55 [1.13:2.13]** | **2.04 [1.44:2.87]** | |

**Table S15**

Membership in each latent class group for parental depression according to personal and family characteristics

| Characteristic | Low ^R^ | High to low | Low to medium | Medium to high | Consistently high | |
| --- | --- | --- | --- | --- | --- | --- |
| *N* (%) | 4358 (78%) | **235 (4%)**** | **451 (8%)**** | **323 (6%)**** | **209 (4%)**** | |
| Parental age | | | | | |  |
| Below average | 2331 (53.5%) | 133 (56.6%) | 225 (49.9%) | **178 (55.1%)*** | 115 (55.0%) | |
| Above average ^R^ | 2027 (46.5%) | 102 (43.4%) | 226 (50.1%) | 145 (44.9%) | 94 (45.0%) | |
| Parent education | | | | | |  |
| Higher ^R^ | 3614 (82.9%) | 187 (79.6%) | 337 (74.7%) | 241 (74.6%) | 134 (64.1%) | |
| Further | 487 (11.2%) | 33 (14.0%) | 64 (14.2%) | **59 (18.3%)*** | **50 (23.9%)*** | |
| Secondary or below | 257 (5.9%) | 15 (6.4%) | **50 (11.1%)*** | 23 (7.1%) | 25 (12.0%) | |
| Single Adult Household | | | | | |  |
| No ^R^ | 3869 (88.8%) | 192 (81.7%) | 352 (78.0%) | 269 (83.3%) | 129 (61.7%) | |
| Yes | 489 (11.2%) | 43 (18.3%) | **99 (22.0%)**** | 54 (16.7%) | **80 (38.3%)**** | |
| Mental Health Diagnosis of Depression/Anxiety/Other | | | | | |  |
| No ^R^ | 3866 (88.7%) | 156 (66.4%) | 300 (66.5%) | 223 (69.0%) | 81 (38.8%) | |
| Yes | 492 (11.3%) | **79 (33.6%)**** | **151 (33.5%)**** | **100 (31.0%)**** | **128 (61.2%)**** | |
| Work |  |  |  |  |  | |
| At home ^R^ | 1415 (32.5%) | 67 (28.5%) | 120 (26.6%) | 87 (26.9%) | 37 (17.7%) | |
| Out of home | 416 (9.5%) | **10 (4.3%)*** | 38 (8.4%) | 32 (9.9%) | 9 (4.3%) | |
| At home and not | 438 (10.1%) | 25 (10.6%) | 37 (8.2%) | 28 (8.7%) | 10 (4.8%) | |
| Not worked | 730 (16.8%) | 52 (22.1%) | **126 (27.9%)*** | 65 (20.1%) | **87 (41.6%)**** | |
| Worked and not | 1359 (31.2%) | 81 (34.5%) | 130 (28.8%) | 111 (34.4%) | 66 (31.6%) | |
|  |  |  |  |  |  | |
| Children's age | | | | | |  |
| < 4 ^R^ | 549 (12.6%) | 32 (13.6%) | 46 (10.2%) | 26 (8.0%) | 19 (9.1%) | |
| 4-10 | 1462 (33.5%) | 84 (35.7%) | 154 (34.1%) | **124 (38.4%)*** | 63 (30.1%) | |
| 11-17 | 848 (19.5%) | 49 (20.9%) | 119 (26.4%) | **77 (23.8%)*** | 54 (25.8%) | |
| Mixed ages | 1499 (34.4%) | 70 (29.8%) | 132 (29.3%) | 96 (29.7%) | 73 (34.9%) | |
| Child SEN/ND Status | | | | | |  |
| No ^R^ | 3849 (88.3%) | 190 (80.9%) | 357 (79.2%) | 255 (78.9%) | 146 (69.9%) | |
| Yes | 509 (11.7%) | **45 (19.1%)*** | **94 (20.8%)*** | **68 (21.1%)*** | **63 (30.1%)**** | |

*Notes.* ^R^ Reference groups; * *p*<.05; ** *p*<.001

Supplementary References

Clark., D. (2022, November 7). UK population 2020, by region. Statista. Retrieved December 12, 2022, from https://www.statista.com/statistics/294729/uk-population-by-region/

GOV.UK. (n.d.). Special educational needs in England, Academic Year 2021/22 – Explore education statistics. Retrieved December 12, 2022, from https://explore-education-statistics.service.gov.uk/find-statistics/special-educational-needs-in-england/2021-22

Kuha, J. (2004). AIC and BIC: Comparisons of Assumptions and Performance. *Sociological Methods & Research*, *33*(2), 188–229.

Lo, Y., Mendell, N. R., & Rubin, D. B. (2001). Testing the number of components in a normal mixture. *Biometrika*, *88*(3), 767–778.

GOV.UK. (2018). Male and female populations. Retrieved December 12, 2022, from https://www.ethnicity-facts-figures.service.gov.uk/uk-population-by-ethnicity/demographics/male-and-female-populations/latest

NHS Digital. (2016, September 29). Adult Psychiatric Morbidity Survey: Survey of Mental Health and Wellbeing, England, 2014. Retrieved December 12, 2022, from https://digital.nhs.uk/data-and-information/publications/statistical/adult-psychiatric-morbidity-survey/adult-psychiatric-morbidity-survey-survey-of-mental-health-and-wellbeing-england-2014

O.E.C.D. Statistics. (n.d.). Educational attainment and labour-force status. Retrieved December 12, 2022, from https://stats.oecd.org/Index.aspx?DataSetCode=EAG_NEAC

Office for National Statistics. (2021a, January 21). Average household income, UK. Retrieved December 12, 2022, from https://www.ons.gov.uk/peoplepopulationandcommunity/personalandhouseholdfinances/incomeandwealth/bulletins/householddisposableincomeandinequality/financialyear2020

Office for National Statistics. (2021b, January 14). Overview of the UK population. Retrieved December 12, 2022, from https://www.ons.gov.uk/peoplepopulationandcommunity/populationandmigration/populationestimates/articles/overviewoftheukpopulation/january2021

Office for National Statistics. (2021c, June 25). Analysis of population estimates tool for UK. Retrieved December 12, 2022, from https://www.ons.gov.uk/peoplepopulationandcommunity/populationandmigration/populationestimates/datasets/analysisofpopulationestimatestoolforuk

Office for National Statistics. (2022a, November 15). A01: Summary of labour market statistics. Retrieved December 12, 2022, from https://www.ons.gov.uk/employmentandlabourmarket/peopleinwork/employmentandemployeetypes/datasets/summaryoflabourmarketstatistics

Office for National Statistics. (2022b). *Ethnic group, England and Wales*. Retrieved December 12, 2022, from https://www.ons.gov.uk/peoplepopulationandcommunity/culturalidentity/ethnicity/bulletins/ethnicgroupenglandandwales/census2021

Vuong, Q. H. (1989). Likelihood Ratio Tests for Model Selection and Non-Nested Hypotheses. *Econometrica*, *57*(2), 307.
